# Curcumin is a Potential Adjuvant to Alleviates Diabetic Retinal Injury *via* Reducing Oxidative Stress and Maintaining Nrf2 Pathway Homeostasis

**DOI:** 10.3389/fphar.2021.796565

**Published:** 2021-12-10

**Authors:** Ting Xie, Xiaodong Chen, Wenyi Chen, Sien Huang, Xinye Peng, Lingmei Tian, Xuejie Wu, Yan Huang

**Affiliations:** ^1^ Department of Ophthalmology and Optometry, Fujian Medical University, Fuzhou, China; ^2^ Medical Technology Experimental Teaching Center, Fujian Medical University, Fuzhou, China; ^3^ Department of Medical Imaging Technology, Fujian Medical University, Fuzhou, China

**Keywords:** diabetic retinopathy, curcumin, oxidative Stress, nuclear factor E2-associated factor 2, transcriptome analysis

## Abstract

Curcumin is a natural polyphenol compound with anti-diabetic, anti-oxidative, and anti-inflammatory effects. Although many studies have reported the protective effect of curcumin in diabetes mellitus or diabetic nephropathy, there is a lack of research on curcumin in diabetic retinopathy. The purpose of this study was to investigate the therapeutic effects of curcumin on the diabetic retinal injury. Streptozotocin (STZ)-induced diabetic rats (60, *n* = 12 each) were respectively given curcumin orally (200 mg/kg/day), insulin subcutaneously (4–6 IU/day), and combined therapy with curcumin and insulin for 4 weeks. Retinal histopathological changes, oxidative stress markers, and transcriptome profiles from each group were observed. Curcumin, insulin, or combination therapy significantly reduced blood glucose, alleviated oxidative stress, and improved pathological damage in diabetic rats. Curcumin not only significantly reduced retinal edema but also had a better anti-photoreceptor apoptosis effect than insulin. In the early stage of diabetes, the enhancement of oxidative stress in the retina induced the adaptive activation of the nuclear factor E2-associated factor 2 (Nrf2) pathway. Treatment of curcumin alleviated the compensatory activation of the Nrf2 pathway induced by oxidative stress, by virtue of its antioxidant ability to transfer hydrogen atoms to free radicals. When curcumin combined with insulin, the effect of maintaining Nrf2 pathway homeostasis in diabetic rats was better than that of insulin alone. Transcriptomic analyses revealed that curcumin either alone, or combined with insulin, inhibited the AGE-RAGE signaling pathway and the extracellular matrix (ECM)-receptor interaction in the diabetic retina. Thus, at the early stage of diabetes, curcumin can be used to alleviate diabetic retinal injury through its anti-oxidative effect. If taking curcumin as a potential complementary therapeutic option in combination with antihyperglycemic agents, which would lead to more effective therapeutic outcomes against diabetic complications.

## Introduction

No matter in developed or developing countries, Diabetes mellitus (DM) has become one of the most severe epidemics in the world (Zimmet et al., 2016). Because of a steep rise in the incidence and prevalence of diabetes in the last decade, diabetic retinopathy (DR), one of the most common and serious microvascular complications of diabetes, has become the main reason for blindness in working-age individuals on a global scale (Chilelli et al., 2013; Gulshan et al., 2016). At present, many factors are thought to be associated with diabetic retinopathy, such as advanced glycation end products, oxidative stress, inflammation, etc. Although the pathophysiology of DR has not been fully elucidated, it has been well documented that oxidative stress plays a crucial role in the occurrence and development of DR (Kowluru, 2013). Due to the characteristics of high oxygen consumption, glucose oxidation, and long-term exposure to light, the retina is highly susceptible to oxidative stress (Catala, 2010; Nabavi et al., 2015a). Under a high glucose environment, the non-enzymatic addition or the glycosylation of monosaccharides (such as glucose) in proteins increases the autooxidation reaction and generates many reactive oxygen species (ROS) (Itoh et al., 1999; Lapolla et al., 2005).

Nuclear factor E2-associated factor 2 (Nrf2) is a critical transcription factor that regulates many antioxidant defense genes. In normal conditions, Kelch-like epichlorohydrin-associated protein-1 (Keap1) binds to the NeH2 domain of Nrf2 to anchor Nrf2 in the cytoplasm, leaving its transcriptional activity dormant (Kensler et al., 2007; Taguchi et al., 2011). However, under stress, Nrf2 dissociates from Keap1 and translocates into the nucleus to activate the transcription of antioxidant defense genes, such as Heme Oxygenase-1 (HO-1) and Superoxide Dismutase (SOD), to reduce the accumulation of ROS. Nox2 is considered as one of the major sources of cytoplasmic ROS in diabetic retinas, where it catalyzes the one-electron reduction of oxygen to superoxide anion and oxidizes cytosolic NADPH. In the initial stage of increased ROS, Nrf2 activation could act as a compensatory mechanism to offset ROS accumulation, but with a continuous increase in ROS, the Nrf2-ARE system will be overwhelmed, resulting in a sustained increase in ROS. Deficiency of Nrf2 could also induce uninhibited activation of Nox2, further exacerbating ROS accumulation (Kowluru and Mishra, 2017). Emerging evidence supports that the transcripts of Nrf2 and Keap1 levels in the diabetic retina are increased, but Nrf2 binds more to Keap1 and less to DNA, which leads to the transcription of the antioxidant enzyme reduced and further compromising the antioxidant defense system (Zhong et al., 2013; Li et al., 2019). In the streptozotocin (STZ) induced diabetes model, Xu et al. show that the absence of Nrf2 in Nrf2^−^/^−^mice elevates the oxidant levels, causes an inflammatory response indicated by elevated levels of TNF-a, increase the vascular permeability of the blood-retinal barrier, compared with Nrf2^+^/^+^mice (Xu et al., 2014). These studies indicate that Nrf2 is a crucial protective factor in the regulation of DR progression, suggesting that heightening the Nrf2 pathway is a potential therapeutic strategy.

Curcumin [5-dione, 1,7-bis (4-hydroxy-3-methoxyphenyl) -6-heptadiene-3], a natural compound, is the main chemical component of *Curcuma longa*. Since curcumin has a wide range of pharmacological activities such as anti-inflammatory, anti-tumor, scavenging active oxidizing substances, lowering blood sugar, etc., its use has found applications in the treatment of diabetes and related complications (Platania et al., 2018). Many animal experiments have confirmed that curcumin can protect oxidative damage cells by mediating Nrf2 antioxidant pathway activation (Zhong et al., 2013). Gonza et al. found that curcumin can protect from heme-induced cerebellar granule neuron injury in rats through activating Nrf2 translocation into the nucleus (González-Reyes et al., 2013). Moreover, Yang et al. found that curcumin can reduce VEGF expression in the retina of diabetic rats and improve the retinal ultrastructural changes caused by diabetes, such as retinal thinning and ganglion cell apoptosis, and disorder of the outer segment of the photoreceptor disc (Yang et al., 2018).

Despite many beneficial effects of curcumin, to our knowledge, few studies have reported the neuroprotective effects in the diabetic retina. Next-generation sequencing-based transcriptome profiling (RNA-seq) analysis is a comprehensive and accurate approach for investigating potential molecular mechanisms underlying complex biological processes (Han et al., 2014) and has been widely applied in diabetic researches (Li et al., 2018). Therefore, this study aims to investigate the protective effect of curcumin on the damaged retina of T1DM rats induced by streptozotocin and use transcriptome sequence-based RNA-seq to identify the differentially expressed genes in retinal tissues of each group, screen the molecular targets, and explore the therapeutic mechanism of curcumin in slowing the progression of diabetic retinopathy.

## Materials and Methods

### Experimental Animals

Seven-week-old male Sprague-Dawley rats, weighing 220–250 g, were purchased from the Animal Experiment Center of Fujian Medical University (Fuzhou, China) and raised in a barrier environment with controlled temperature (20–25°C), humidity (50 ± 5%) and lighting (12 h light/dark cycle). Five groups (*n* = 12 each) as follows: Group 1: control rats treated with vehicle (CON); Group 2: diabetic model rat (DM); Group 3: diabetic rats treated with curcumin (CUR); Group 4: diabetic rats treated with insulin (INS); Group 5: diabetic rats treated with curcumin and insulin (CUR + INS). Except for the control group, rats from other groups were rendered diabetic by a single intravenous injection of 55 mg/kg streptozotocin (STZ). Three days after STZ injection, fasting blood was collected from the tail vein and measured with a glucose meter (Roche Inc., Basel, Switzerland). Rat with glucose levels >16.7 mmol/L, polyuria, was defined as diabetic and included in the experiments.

### Treatment Protocols

Drug intervention began after the successful establishment of the diabetic model. Rats in the CUR group were dosed by oral gavage once per day for 4 weeks with curcumin (CAS: 458-37-7; Sigma, Saint Louis, MO, United States) at dosages of 200 mg/kg in a volume of 1% sodium carboxymethyl cellulose diluted. The other groups received the same amount of vehicle via gavage. Rats in the (INS) group and (CUR + INS) group were given a subcutaneous injection of long-acting insulin on the neck and back, with a total dose of 4–6 IU/day. Start with a small dose and gradually increase to the most appropriate dose (blood glucose could be maintained at 6–10 mmol/L). The blood glucose was measured every 2 days on average to adjust the insulin dosage. At the end of the 4-weeks treatment, animals were weighed, fasted overnight, and anesthetized using an intraperitoneal injection of 3% sodium pentobarbital (50 mg/kg). While under anesthesia, they were painlessly sacrificed. The rat eyes from each group were removed, and the retina was isolated.

### Biochemical Assay

The intracellular activity of superoxide dismutase (SOD; Cat. No. BC0175) and malondialdehyde (MDA; Cat. No. BC0025) were measured by commercially available assay kits purchased from Solarbio Science and Technology Co., Ltd. (Beijing, China). The levels of total glutathione (T-GSH), oxidized glutathione (GSSG), and reduced glutathione (GSH) were estimated using a kit (Cat. No. A061-1-1) from Jiancheng Bioengineering Institute (Nanjing, China). All procedures were in accordance with standard protocols described in the kits. Optical density (OD) values were measured by thermo scientific microplate reader (Thermo Fisher Scientific, Waltham, MA, United States). All assays were carried out in triplicates.

### Haematoxylin and Eosin Staining

After 4 weeks, eyes were enucleated upon euthanasia and processed for histopathology, including fixation overnight in FAS eye fixation fluid followed by dehydration in graded ethanol, embedding in paraffin, and hematoxylin and eosin (H&E) staining by a series of standard techniques. The cross-section slice of the retina was selected from each eye in which optic nerve head area could be found when observed the retinal layer under a microscope. Paraffin slice thickness was set at 5 μm. The images were taken from the same part of the retina layer and saved using imaging software (DS-U3 imaging system, Nikon Instrument Inc., Japan). The total retina thickness was measured by the CaseViewer 2.3 analysis system (Servicebio Biotechnology Co. Ltd., Wuhan, China). Three sections per eye were averaged in five distinct areas of ×400 fields, and there were three rats in each group.

### Terminal Deoxynucleotidyl Transferase dUTP Nick End Labelling Staining

The paraffin section was used to evaluate the apoptosis of retinal cells in different groups by detecting the DNA fragmentation in the nucleus. Staining was performed using the fluorescein (FITC) TUNEL cell apoptosis detection kit (Cat. No. G1501, Servicebio Biotechnology Co. Ltd., Wuhan, China) according to the manufacturer’s instructions. Firstly, the tissue was covered with proteinase K working solution at 37°C for 25 min and followed by adding 0.1% triton solution to break the membrane at room temperature for 20 min. Secondly, tissue was incubated with terminal deoxynucleotidyl transferase (TDT) enzyme reaction solution (TDT enzyme, dUTP, and buffer at 1:5:50 ratio) for 2 h at 37°C. Each section was washed three times with phosphate-buffered saline (PBS, pH = 7.4) in a Rocker device, 5 min each. Finally, using the anti-fluorescence quenching agent containing 4,6-diamino-2-phenyl indole (DAPI) for 15 min at room temperature to seal the sections. The retinas from each group were observed under Ortho-Fluorescent Microscopy. (Nikon Eclipse C1, Nikon Instrument Inc., Japan). TUNEL-positive cells were calculated in five random fields per retina and averaged. Retinal cell apoptosis rate = TUNEL positive cell number/normal cell number.

### Protein Extraction and Western Blot Analyses

The proteins from retinal tissues were extracted using radioimmunoprecipitation assay lysis buffer with protease inhibitor phenylmethylsulfonyl fluoride. After incubation for 30 min on ice, lysates were centrifuged at 13,800 g for 15 min at 4°C, and the supernatants were collected. Before immunoblotting, the protein concentration of each sample was determined using the Enhanced BCA Protein Assay kit (Beyotime Biotechnology Co. Ltd., Shanghai, China) to ensure equal loading among lanes. Protein samples were boiled in SDS-PAGE sample loading buffer for 10 min, and an equal amount of protein (40 µg/well) was loaded in each well of 10–15% SDS-polyacrylamide gels and separated electrophoretically at 120 V for 80 min. Separated proteins were then electrophoretically transferred onto polyvinylidene difluoride (PVDF) membranes (Merck Millipore, Darmstadt, Germany) at 100 V for 1 h and blocked with 5% non-fat milk made in Tris-buffered saline containing 0.1% Tween-20 (TBS-T) for 2 h at room temperature. After blocking, the membranes were rinsed three times with TBS-T buffer for 5 min each and incubated overnight at 4°C with anti-β-actin (1:3,000; Beyotime Biotechnology Co. Ltd., Shanghai, China), anti-Nrf2 (1:1,000; ImmunoWay Biotechnology Inc., Plano, TX, United States), anti-HO-1 (1:2,000; Abcam, Cambridge Science Park, England), anti-p62 (1:1,000; ImmunoWay Biotechnology Inc., Plano, TX, United States), anti-microtubule-associated protein light chain 3 (LC3, 1:1,000; Proteintech Group, Inc., Chicago, IL, United States), primary antibodies.

After three 7 min washes in Tris-buffered saline with TBS-T buffer, membranes were incubated with the horseradish peroxidase-conjugated secondary antibodies (1:5,000; ImmunoWay Biotechnology Inc., Plano, TX, United States) at room temperature for 60 min. With the ECL advance Western Blotting detection kit (Meilune Biotechnology Co. Ltd., Dalian, China), the immunoreactivity of bands was visualized on a Molecular Imager Gel Image System with Image Lab software (Bio-Rad Laboratories, Inc., Hercules, CAL., United States) and the gray value of bands were analyzed by ImageJ software (Bethesda, United States). β-actin was set as the loading control.

### Immunofluorescence Staining

Paraffin-embedded retina tissue sections were deparaffinized, hydrated, microwaved in Tris-EDTA buffer for antigen retrieval, and finally rinsed in PBS. After blocking with bovine serum albumin for 30 min to block endogenous peroxidase activity, the sections were incubated overnight with the rabbit anti-Nrf2 (1:200, Servicebio Biotechnology Co. Ltd., Wuhan, China). The negative control was incubated with PBS. After adequate washing, sections were coincubated with goat anti-rabbit IgG H&L (1:1,500, Servicebio Biotechnology Co. Ltd., Wuhan, China) for 2 h at room temperature and then stained with DAPI for 5 min. Images were acquired at 400× magnification using Ortho-Fluorescent Microscopy. (Nikon Eclipse C1, Nikon Instrument Inc., Japan) and five fields of view were randomly selected for each sample.

### RNA Extraction, Library Preparation, and Sequencing

Total RNA was extracted from the retina using RNAprep Pure Tissue kit purchased from TianGen Biotechnology Inc. (Beijing, China) according to the manufacturer’s instructions. RNA degradation and contamination were monitored on 1% agarose gels, as shown in Figure 4A. RNA concentration was measured with a Qubit 2.0 fluorometer. Subsequently, the SMART-RNAseq Library Index kit (Kaitai Biotechnology Co. Ltd., Hangzhou, China) was used to isolate poly(A)^+^ mRNA from the total RNA and then fragmented. The single-stranded oligonucleotide was used to synthesize cDNA, which was amplified and enriched by LightCycler® 96 Real-Time PCR System (Roche Inc., Basel, Switzerland) to prepare sequencing libraries. Finally, all samples were sequenced on an Illumina Novaseq 6000® System (Illumina Inc., San Diego, CAL., United States) as per the corresponding user guide, and 150-bp paired-end reads were generated.

### Sequencing Data Processing and Analyzing

To obtain clean reads, fastp software (https://github.com/OpenGene/fastp) was used to remove the adaptor and the RNA-seq reads with the length less than 50 bp or with poly(N) (>5 base) or low-quality bases (quality value less than 20 bp). The clean reads were then aligned to the Ensemble rat reference genome (Rnor 6.0) using bowtie2 (http://bowtiebio.sourceforge.net/bowtie2/index.shtml). Subsequently, stringtie software (https://ccb.jhu.edu/software/stringtie/) was used to assemble the transcripts. Differential express genes (DEGs) were identified using DESeq2 (https://bioconduc-tor.org/packages/release/bioc/html/DESeq2.html) with default parameters and the significance threshold (FDR < 0.05 and |log2 (fold change)| > 1). The selected DEGs were subjected to the R package clusterProfiler for gene ontology (GO) and Kyoto Encyclopedia of Genes and Genomes (KEGG) pathway analysis. The significant enrichment of DEGs was determined by an adjusted *p*-value < 0.05. The different expression of candidate genes was verified by quantitative real-time PCR (qPCR).

### Quantitative Real-Time Polymerase Chain Reaction Analysis

Total RNA was extracted from the retina of each group using the Trizol Reagent (Thermo Fisher Scientific, Waltham, MA, United States). Subsequently, RNA was re-verse-transcribed to cDNA using Hifair® Ⅲ first Strand cDNA Synthesis SuperMix kit (Yeasen Biotechnology Co. Ltd., Shanghai, China) following the manufacturer’s instructions. The reaction of real-time PCR was performed in Hieff ® qPCR SYBR Green Master Mix (Yeasen Biotechnology Co. Ltd., Shanghai, China), which contained cDNA 1.2 µL (800 ng/μL), SYBR green mix 10 μL, 0.4 µL each forward or reverse primer (2 μmol L^−1^), 8 µL distilled water. The primer pairs were designed by Beacon Designer 7 as per the published gene sequences of the NCBI database. Real-time PCR was performed using a System 7,500 instrument (Applied Biosystems, Carlsbad, CA, United States), and the following thermocycling conditions were as follows: 95°C pre-denaturation for 5 min, followed by 40 cycles at 95°C for 10 s, 60°C for 35 s, 95°C for 15 s. After the reactions, melt curve analysis was used to assess whether the intercalating dye qPCR assays had produced single, specific products. Relative expression of target genes was normalized to β-actin and calculated according to the 2^−∆∆Ct^ method (Livak and Schmittgen, 2001). Each sample was analyzed in triplicate. All primers are listed in Supplementary Table S5.

### Statistical Analysis

All the results were expressed as mean ± standard deviation (SD). SPSS software version 23.0 (IBM, Inc., Armonk, NY, United States) was used to perform all statistical analyses of the physiological indexes. Differences between the groups were determined by one-way analysis of variance (ANOVA) with LSD-t *post-hoc* multiple comparison test. Probability values <0.05 were considered significant.

## Results

### Effects of Curcumin on Body Weight and Body Glucose in All Groups

Table 1 showed the physical characteristics during the four-week intervention. At the beginning of the study, there were no statistically significant differences between any two groups regarding the fasting body weight (FBW) and fasting blood glucose (FBG). The injection of rats with STZ resulted in a significant increase in blood glucose levels as compared with the control group (^**^ *p* < 0.01), which showed symptoms of polyuria, polydipsia, and polyphagia. By the end of the experiment, the diabetic rats from the DM group exhibited lower body weights than the rats from the CON group (^**^ *p* < 0.01). After 4 weeks of intervention, insulin alone or combined with curcumin was able to significantly reduce hyperglycemia (*p* < 0.01) and weight loss (*p* < 0.01) in diabetic rats, in addition to improving polyphagia, polydipsia, and polyuria. It should be noted that rats in the INS group showed slight polyphagia, polydipsia 5–7 h after insulin injection, which became worse gradually with the metabolism of insulin. Unlike the INS group, the food intake and water intake of diabetic rats in (CUR + INS) group had no significant fluctuation within 24 h of administration. However, although the results showed that curcumin alone had partly effects on reducing blood glucose (^##^ *p* < 0.01) and alleviating the symptom of polyphagia, polydipsia, and polyuria, it did not significantly improve weight loss in diabetic rats.

**TABLE 1:** Effects of treatment on fasting blood glucose and body weight in experimental animals.

Group	Fasting blood glucose (mmol·L^−1^)			Fasting body weight (g)		
	Initial	72 h	4 weeks	Initial	72 h	4 weeks
CON	4.5 ± 0.4	4.3 ± 0.4	5.5 ± 0.6	230.8 ± 10.0	267.8 ± 16.6	412.8 ± 23.8
DM	4.4 ± 0.5	30.2 ± 3.8^**^	23.3 ± 4.2^**^	246.7 ± 9.4	257.5 ± 25.1	298.5 ± 60.0^**^
CUR	4.6 ± 0.5	24.9 ± 5.3^**^	13.5 ± 8.2^##^	249.5 ± 9.7	262.5 ± 12.8	315.0 ± 45.0^**^
INS	4.3 ± 0.6	27.2 ± 5.7^**^	7.2 ± 2.1^##^	249.0 ± 10.4	258.0 ± 11.5	381.5 ± 14.9^##, &&^
CUR + INS	4.5 ± 0.5	25.5 ± 7.3^**^	7.8 ± 2.8^##^	245.0 ± 14.6	254.2 ± 11.3	349.8 ± 55.0^##, &^

CON, control group; DM, STZ-induced diabetic model group; CUR, 200 mg/kg curcumin treatment group; INS, long-acting insulin treatment group; CUR + INS, 200 mg/kg curcumin and long-acting insulin treatment group. All data are expressed as the mean ± standard deviation (*n* = 3). ^**^ *p* < 0.01 vs. the CON group; ^##^ *p* < 0.01 vs. the DM group; ^&&^ *p* < 0.01 vs. the CUR group; ^&^ *p* < 0.05 vs. the CUR group.

### Effects of Curcumin on Oxidative Stress of Retina in STZ-Induced Diabetic Rats

The retinal content of MDA, a biomarker of oxidative stress, and antioxidant enzymes (such as SOD, T-GSH, GSSG, and GSH) was measured to investigate the effect of curcumin on oxidative damage of the retina induced by hyperglycemia (Table 2). Concerning retinal oxidative stress indices, the DM group showed significantly elevated levels in the MDA concentration and a higher ratio of GSSG/GSH (^**^ *p* < 0.01), but the activities of SOD and T-GSH were significantly lower than those in the CON group (^**^ *p* < 0.01). Unexpectedly, although curcumin treatment significantly reduced MDA level compared with the DM group (^##^ *p* < 0.01), it still failed to increase the activity of SOD and the other antioxidant enzymes (no statistical difference compared with the DM group). A combination treatment of curcumin and insulin was more effective than curcumin alone, not only in reducing MDA level and the ratio of GSSH to GSH but also in increasing the content of the SOD and T-GSH (^##^ *p* < 0.01). Moreover, intervention with insulin successfully restored the activities of these enzymes and significantly reduced oxidative stress of the diabetic rats (^**, ##^ *p* < 0.01).

**TABLE 2:** Effects of treatment on retinal superoxide dismutase, malondialdehyde, and glutathione content of homogenate of retinal tissues.

Group	SOD (U·g^−1^)	MDA (nmol·g^−1^)	T-GSH (µmol·L^−1^)	GSSG/GSH
CON	47.19 ± 3.3	35.56 ± 0.8	2.97 ± 0.7	0.96 ± 0.0
DM	30.99 ± 2.9^**^	54.93 ± 0.4^**^	1.31 ± 0.1^**^	1.31 ± 0.0^**^
CUR	26.88 ± 2.1^**, ##^	31.57 ± 0.7^**, ##^	1.04 ± 0.1^**, ##^	1.52 ± 0.1^**, ##^
INS	61.10 ± 2.5^**, ##, &&^	36.30 ± 0.6^##, &&^	2.90 ± 0.0^##, &&^	0.97 ± 0.0^##, &&^
CUR + INS	41.27 ± 1.7^**, ##, &&^	35.94 ± 1.2^##, &&^	1.91 ± 0.0^**, ##, &&^	1.10 ± 0.0^##, &&^

CON, control group; DM, STZ-induced diabetic model group; CUR, 200 mg/kg curcumin treatment group; INS, long-acting insulin treatment group; CUR + INS, 200 mg/kg curcumin and long-acting insulin treatment group; SOD, superoxide dismutase; MDA, malondialdehyde T-GSH, total glutathione; GSSG, oxidized glutathione; GSH, reduced glutathione. All data are expressed as the mean ± standard deviation (*n* = 3). ^**^ *p* < 0.01 vs. the CON group; ^##^ *p* < 0.01 vs. the DM group; ^&&^ *p* < 0.01 vs. the CUR group.

### Effects of Curcumin on Retinal Morphology in STZ-Induced Diabetic Rats

**FIGURE 1:**
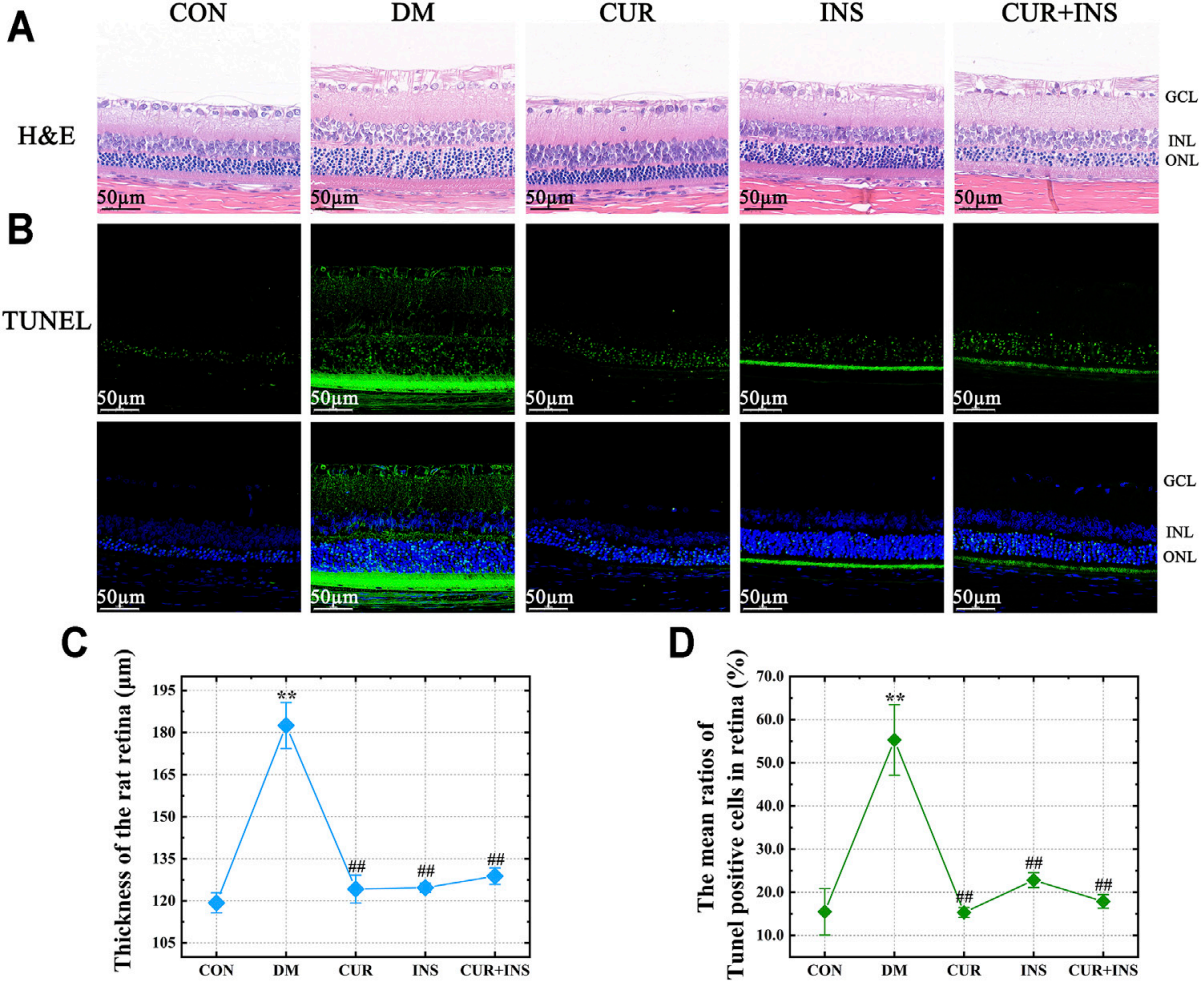
Effect of curcumin on the morphology and cell apoptosis of the retinas in diabetic rats after 4 weeks treatment. Representative images of **(A)** H&E-stained sections (bar = 50 µm) and **(B)** TUNEL-stained sections (bar = 50 µm) of the retinal tissues among different groups; **(C)** The thickness of the overall retina in different groups; **(D)** The mean ratios of TUNEL positive cells of the rat retinas among different groups. CON, control group; DM, STZ-induced diabetic model group; CUR, 200 mg/kg curcumin treatment group; INS, long-acting insulin treatment group; CUR + INS, 200 mg/kg curcumin and long-acting insulin treatment group; GCL, ganglion cell layer; INL, inner nuclear layer; ONL, outer nuclear layer. All data are expressed as the mean ± standard deviation (*n* > 3). ^**^ *p* < 0.01 vs. the CON group; ^##^ *p* < 0.01 vs. the DM group.

The morphological changes in the retina of rats are shown in Figure 1. H&E staining showed that there were no noticeable pathological changes in the retina of the CON group, and each layer of the retina was arranged neatly and presented a clear and complete structure. However, in the DM group, although the structural layers of the retina were still evident, the thickness of retinal tissue markedly increased because of edema (^**^ *p* < 0.01) (Figures 1A,C). Moreover, the cells in each retinal layer of the diabetic rat were arranged loosely, the inner nuclear layer presented severe vacuolation, and the cell density in the outer nuclear layer was increased (Figure 1A). After treatment with curcumin, the diabetes-induced retinal thickening was mitigated (^##^ *p* < 0.01) (Figure 1C), and there were no remarkable retinal abnormalities in the CUR group, as retinal cells were well-shaped, closely arranged in each layer, and the cell density of the inner and outer nuclear layer was increased compared with the DM group. In the INS group, although abnormalities edema and disarrangement were less than that in the DM group, there was still mild edema in the outer nuclear layer and individual vacuolation in the inner nuclear. The retina of the (CUR + INS) group exhibited a normal arrangement of each retinal cell layer. These results indicate that curcumin has an excellent protective effect on retinal morphological damage caused by diabetes (Figure 1A).

### Effects of Curcumin in Neuronal Apoptosis in STZ-Induced Diabetic Rats

Terminal deoxynucleotidyl transferase dUTP nick end labeling (TUNEL) staining was used to estimate the degree of apoptosis in the retina. A few TUNEL-positive cells were detected in the CON group (Figure 1B). whereas the ratios of TUNEL-positive neurons in the retinas of diabetic rats compared with the CON group increased from 15.5 ± 5% to 55.3 ± 8% (^**^ *p* < 0.01) (Figure 1D). Moreover, the location of the TUNEL-positive nuclei in the DM group was mainly in the inner nuclear and photoreceptor layer (Figure 1B). The results also suggested that all the drug intervention groups, including the CUR group, INS group, and (CUR + INS) group, exert an excellent anti-apoptotic effect in early diabetic retinas (^##^ *p* < 0.01). It is worth noting that intervention with insulin did not significantly reduce the number of apoptosis cells in the retinal photoreceptor layer (Figure 1B).

### Effects of Curcumin on Nrf2 Signaling-Related Genes and Proteins Expression Levels in STZ-Induced Diabetic Rats

**FIGURE 2:**
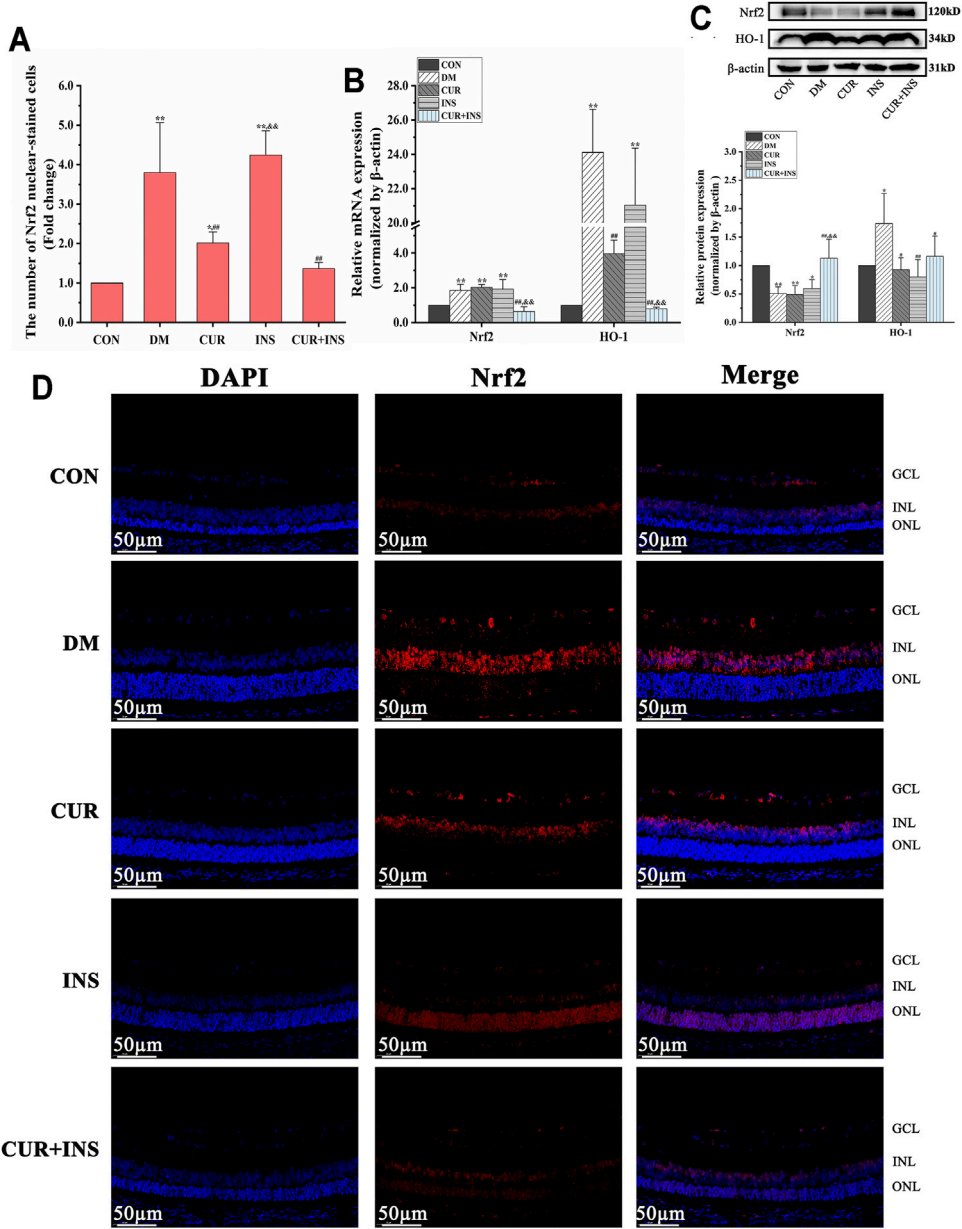
Effects of treatment on the expression levels of Nrf2 pathway in the retina of rats. **(A)** The Nrf2 nuclear-stained cells in retinas of rats among different groups. **(B)** The relative mRNA expression levels of Nrf2 and HO-1. **(C)** Quantification of immunoblots of Nrf2 and HO-1 **(D)** Nrf2 immunofluorescence staining sections (bar = 50 µm). CON, control group; DM, STZ-induced diabetic model group; CUR, 200 mg/kg curcumin treatment group; INS, long-acting insulin treatment group; CUR + INS, 200 mg/kg curcumin and long-acting insulin treatment group. All data are expressed as the mean ± standard deviation (*n* > 3). ^**^ *p* < 0.01 vs. the CON group; ^*^ *p* < 0.05 vs. the CON group; ^##^ *p* < 0.01 vs. the DM group; ^#^ *p* < 0.05 vs. the DM group; ^&&^ *p* < 0.01 vs. the CUR group.

As shown in Figure 2, high glucose caused 1.9-fold and 24.1-fold increase in retinal Nrf2 and HO-1 mRNA levels, respectively, compared to the levels seen in the control group (^**^ *p* < 0.01). Western blot analysis revealed that the HO-1 protein level of the DM group increased (^*^ *p* < 0.05) but total Nrf2 protein decreased (^**^ *p* < 0.01). Moreover, Immunofluorescence showed that the number of Nrf2 nuclear-stained cells increased significantly in the DM group, compared with the CON group (^**^ *p* < 0.01). These results suggested that oxidative stress induced by hyperglycemia increased Nrf2 pathway activity in diabetic retinas. After curcumin intervention, although the total protein content and mRNA level of Nrf2 were not significantly different from that of the DM group, the expression level of HO-1 protein and the number of Nrf2 nuclear-stained cells were significantly decreased. This indicated that the activation of the Nrf2 pathway was restricted. Furthermore, the activation state of the Nrf2 pathway in the retina of diabetic rats was completely reversed by combination therapy of curcumin and insulin. The mRNA or protein level of Nrf2 and HO-1 and the number of Nrf2 nuclear-stained cells in the (CUR + INS) group showed no significant difference compared with the CON group. However, there were no significant differences between the INS group and DM group in the mRNA levels of HO-1 and Nrf2, the total protein level of Nrf2, and the number of Nrf2 nuclear-stained cells, but HO-1 protein expression of the INS group was lower than that in DM group (^**^ *p* < 0.01).

### Effects of Curcumin on Autophagy Proteins Expression Levels in STZ-Induced Diabetic Rats

**FIGURE 3:**
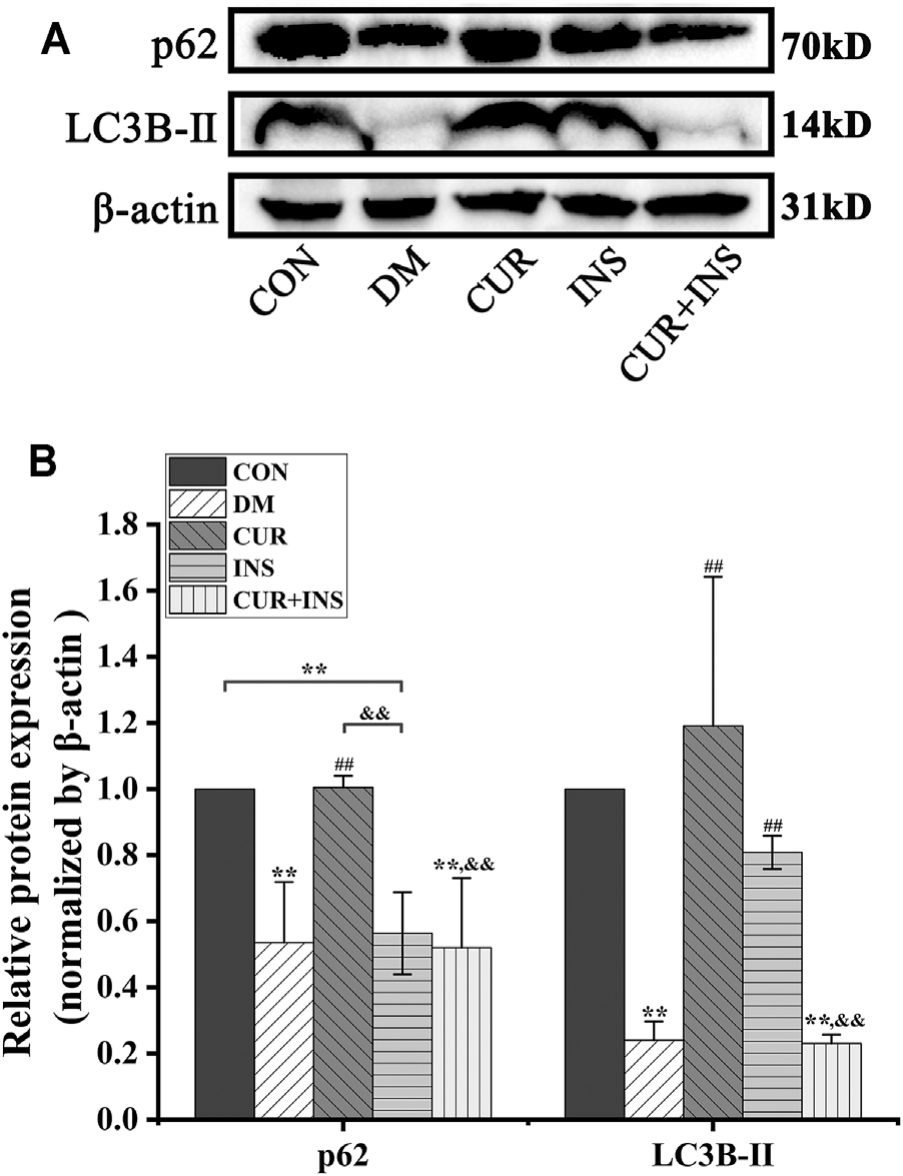
Effects of treatment on the relative protein expression levels of p62 and LC3B-Ⅱ in the retina of rats. **(A)** Representative immunoblots of p62 and LC3B-Ⅱ in retinas of rats among different groups. **(B)** Quantification of immunoblots of p62 and LC3B-Ⅱ, β-actin used as the loading control. CON, control group; DM, STZ-induced diabetic model group; CUR, 200 mg/kg curcumin treatment group; INS, long-acting insulin treatment group; CUR + INS, 200 mg/kg curcumin and long-acting insulin treatment group. All data are expressed as the mean ± standard deviation (*n* > 3). ^**^ *p* < 0.01 vs. the CON group; ^##^ *p* < 0.01 vs. the DM group; ^&&^ *p* < 0.01 vs. the CUR group.

Western blotting analysis showed a significant reduction in the expression level of p62 and LC3B-Ⅱ in the retina of diabetic rats compared to the CON group (^**^ *p* < 0.01), which suggested that high glucose decreased retinal autophagy. However, after the curcumin supplementary, the expression of p62 and LC3B-Ⅱ protein markedly accumulated in the diabetic retina (^##^ *p* < 0.01). In the INS group, Insulin treatment maintained the p62 and significantly increased LC3B-Ⅱ protein expression level (^##^ *p* < 0.01) relative to those in the DM group. Surprisingly, the effect of combination therapy on autophagy in diabetic rats was quite different from that of curcumin or insulin alone. The results showed that the combination therapy of curcumin and insulin did not increase the expression levels of p62 and LC3B-Ⅱ proteins in the retina of diabetic rats (No statistical significance compared with the DM group) (Figure 3).

### Acquisition and Analysis of Differentially Expressed Genes

**FIGURE 4:**
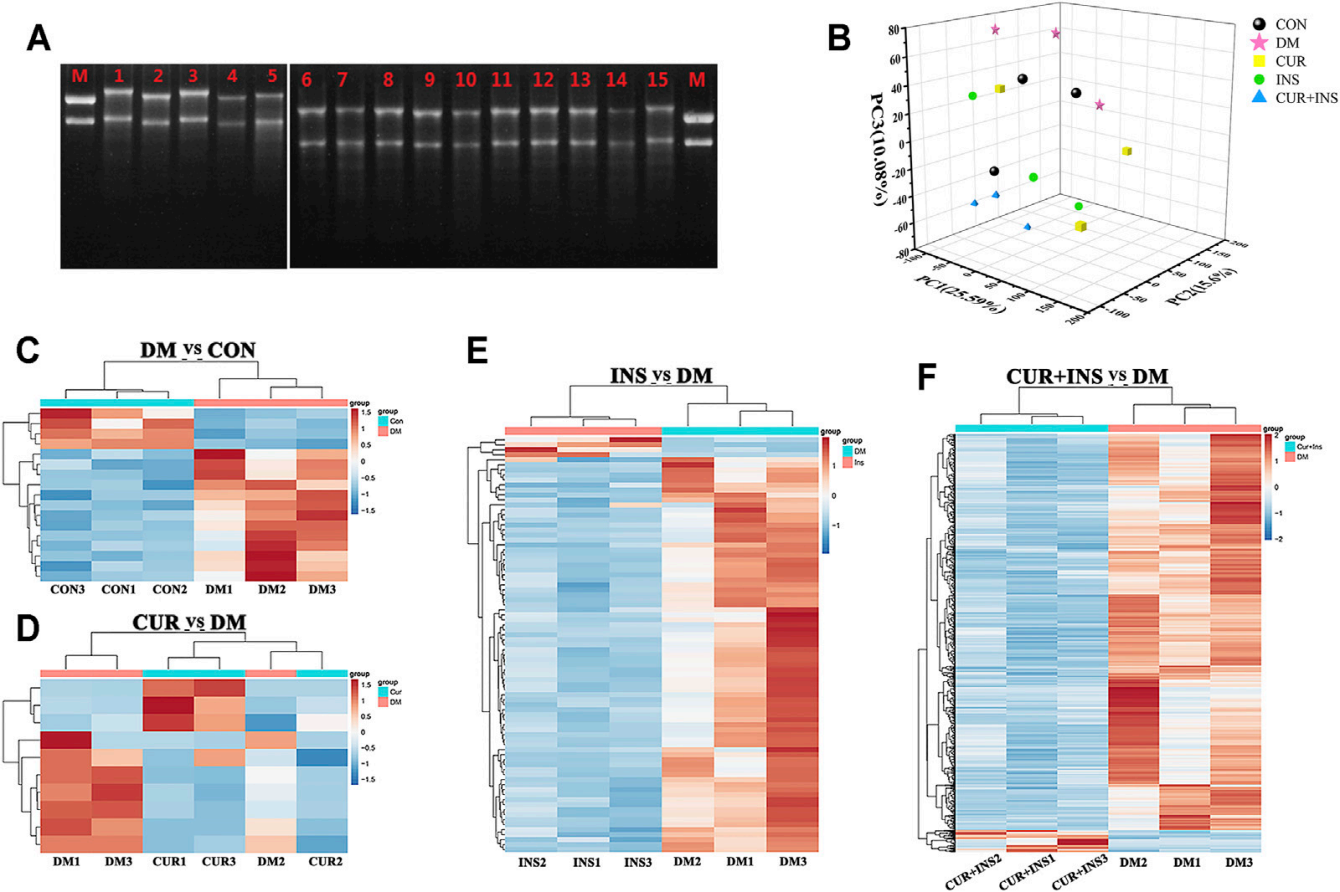
Transcriptome Profiling Analysis for the rat retina across the control (CON), diabetic model rat (DM), 200 mg/kg curcumin treatment (CUR), long-acting insulin treatment (INS), and 200 mg/kg curcumin and long-acting insulin treatment (CUR + INS) groups. **(A)** Using the 1% agarose gel electrophoresis to examine the integrity of RNA in different groups. Lane M: RNA marker; Lanes 1, 2, and 3: three CON samples; Lanes 4, 5, and 6: three DM samples; Lanes 7, 8, and 9: three CUR samples; Lanes 10, 11, and 12: three INS samples; Lanes 13, 14 and 15: three (CUR + INS) samples. **(B)** the three-dimensional scattered plots of the principal component analysis, which indicated a distinct separation among five different groups. PC, principal component. Heat maps showing the whole differentially expressed genes from the DM-vs-CON **(C)**, CUR-vs-DM **(D)**, INS-vs-DM **(E)**, and (CUR + INS)-vs-DM **(F)** comparisons. Each column represents an individual retinal sample. Red indicates upregulated, and blue downregulated genes.

To investigate the potential molecular mechanism of curcumin in improving diabetic retinopathy, we further investigated the gene expression profiles of retinal tissues in different groups of rats by RNA-Seq. The results of agarose gel electrophoresis showed that the total RNA extracted from each sample had good integrity and purity for library construction (Figure 4A). The raw data were filtered by fastp software to obtain clean data to ensure the reliability of the library. As shown in Table 3, we obtained approximately 37.1–50.7 million high-quality clean reads from each group, and the total clean reads Q30% exceeded 95.61%. These results indicated that the quality of all libraries was favorable and suitable for subsequent analysis.

**TABLE 3:** Assessment of assembly quality of cDNA libraries in different groups.

Sample	Raw reads	Clean reads	Error rate (%)	Q30 (%)
CON_1	46,131,466	45,241,610	1.93	95.61
CON_2	51,569,770	50,658,160	1.77	95.73
CON_3	48,429,196	47,392,902	2.14	95.8
DM_1	42,501,918	41,664,424	1.97	95.81
DM_2	42,638,760	41,851,520	1.85	96.02
DM_3	48,243,474	47,324,528	1.90	96.01
CUR_1	45,242,892	44,354,994	1.96	96.06
CUR_2	44,205,066	43,278,154	2.10	95.99
CUR_3	45,319,262	44,238,482	2.38	96.08
INS_1	38,022,604	37,119,670	2.37	95.95
INS_2	46,291,590	45,419,474	1.88	96.04
INS_3	44,992,870	44,104,324	1.97	95.72
CUR + INS_1	39,203,986	38,397,162	2.06	95.86
CUR + INS_2	39,944,208	38,875,372	2.68	96.00
CUR + INS_3	43,483,738	42,590,536	2.05	95.97

CON, control group; DM, STZ-induced diabetic model group; CUR, 200 mg/kg curcumin treatment group; INS, long-acting insulin treatment group; CUR + INS, 200 mg/kg curcumin and long-acting insulin treatment group.

The principal component analysis (PCA) was performed on the normalized expression values of the genes to observe a separation of distinct clusters for the different groups. The contribution ratios of the first, second, and third principal components are 25.59, 15.6, and 10.08%, respectively. As shown in the 3D scatter of PCR (Figure 4B), the principal component clusters of any two groups were clearly separated, suggesting that the transcripts of diabetic rats did have changed, and the expression of genes varies under three varying treatments regimens. Hierarchical clustering analysis of DEGs was shown in Figure 4C, which was used to identify differentially expressed genes in the four comparisons: DM/CON, CUR/DM, INS/DM, and (CUR + INS)/DM. For the DM group, 17 DEGs were identified relative to the CON group (13 up-regulated and 4 down-regulated; Figure 4C). In the CUR/DM comparison, there were only 10 DEGs (3 up-regulated and 7 down-regulated; Figure 4D). For the INS group, 90 differentially expressed genes (4 up-regulated and 86 down-regulated) were identified relative to the DM group. Notably, compared with the DM group, the combination therapy of curcumin and insulin significantly altered the expression of 747 genes. Among them, 708 genes were down-regulated, and 39 genes were up-regulated (Figure 4E). The complete lists of the differentially expressed genes are provided in Supplementary Table S2–S5. To sum up, the results indicated that curcumin used alone or combined with insulin did change the retinal gene expressions of diabetic rats.

### Biological Function and Pathway Analyses of Differentially Expressed Genes

**FIGURE 5:**
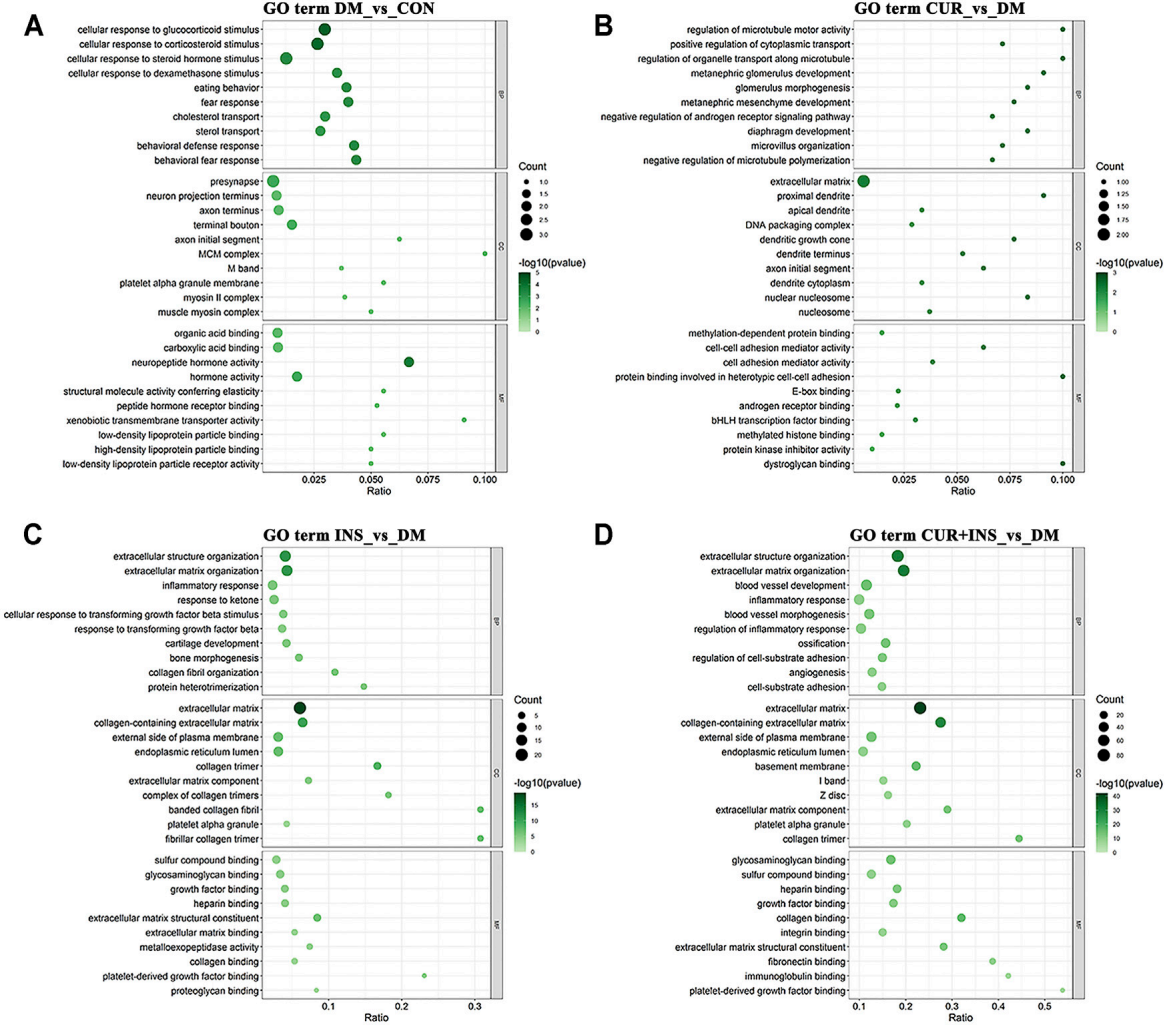
Top 10 most significantly Gene Ontology (GO) functional enrichment analysis terms of differentially expressed genes in three types of functions: biological process (BP), cellular component (CC), and molecular function (MF). Bubble diagram showing the enriched GO results from the DM-vs-CON **(A)**, CUR-vs-DM **(B)**, INS-vs-DM **(C)**, and (CUR + INS)-vs-DM **(D)** comparisons. Y-axis: the significantly GO category entries; X-axis: the Ratio of the number of genes enriched by differentially expressed genes to the GO item and the number of genes enriched by all genes to the GO item. The color of the bubble represents the enrichment significance *p*-value, and the size of the bubble represents the number of differentially expressed genes.

**FIGURE 6:**
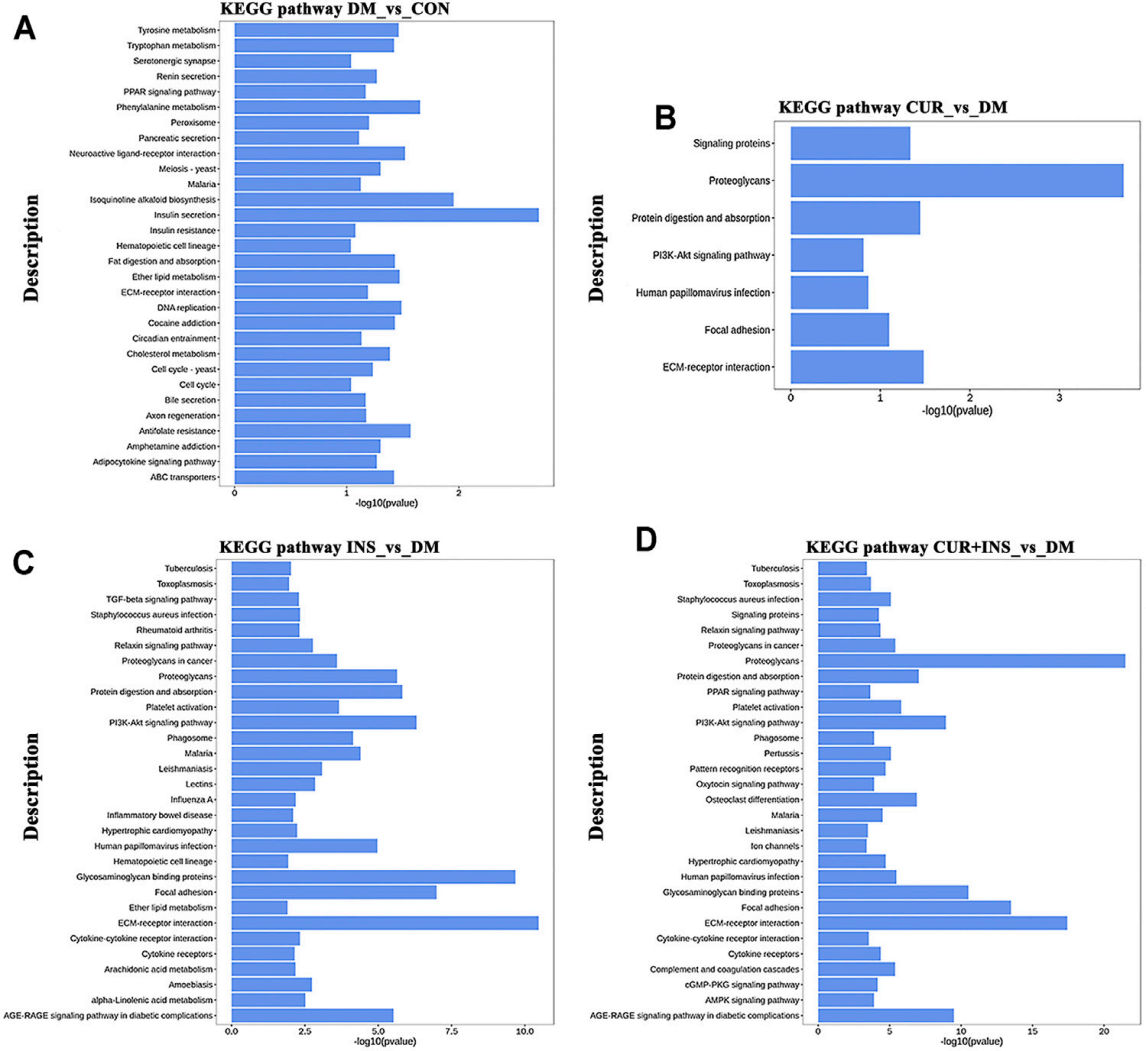
Top 30 most significantly enriched KEGG pathways of differentially expressed genes from the DM-vs-CON **(A)**, CUR-vs-DM **(B)**, INS-vs-DM **(C)**, and (CUR + INS)-vs-DM **(D)** comparisons. Y-axis: the significantly KEGG pathways; X-axis: the significance [−log10 (*p*-value)] of DEG-enriched pathways.

The Gene Ontology (GO) analysis and Kyoto Encyclopedia of Genes and Genomes (KEGG) pathway analysis were performed to investigate further the functional associations of the differentially expressed genes (Figure 5, 6). According to the GO results, there are three types of functions: biological process, cellular component, and molecular function. Compared with the CON group, the most predominant GO terms of the DM group in the biological process were dominated by cellular responses to hormonal stimulation such as glucocorticoid and corticosteroid, and the most affected molecular function was still associated with hormone activity such as neuropeptide hormone activity. The primary cellular component affected by high blood glucose included the presynapse and terminal bouton (Figure 5A). The GO analysis for the CUR vs. DM differentially expressed genes indicated significant enrichment of gene function related to the regulation of microtubule motor activity, protein binding involved in heterotypic cell-cell adhesion, and the cellular component of the proximal dendrite (Figure 5B). Interestingly, between the INS vs. DM comparison and the (CUR + INS) vs. DM comparison, except for the molecular function, the top three significantly enriched GO terms in the other two functions were the same, which were related to extracellular structure organization and extracellular matrix (Figures 5C,D).

**FIGURE 7:**
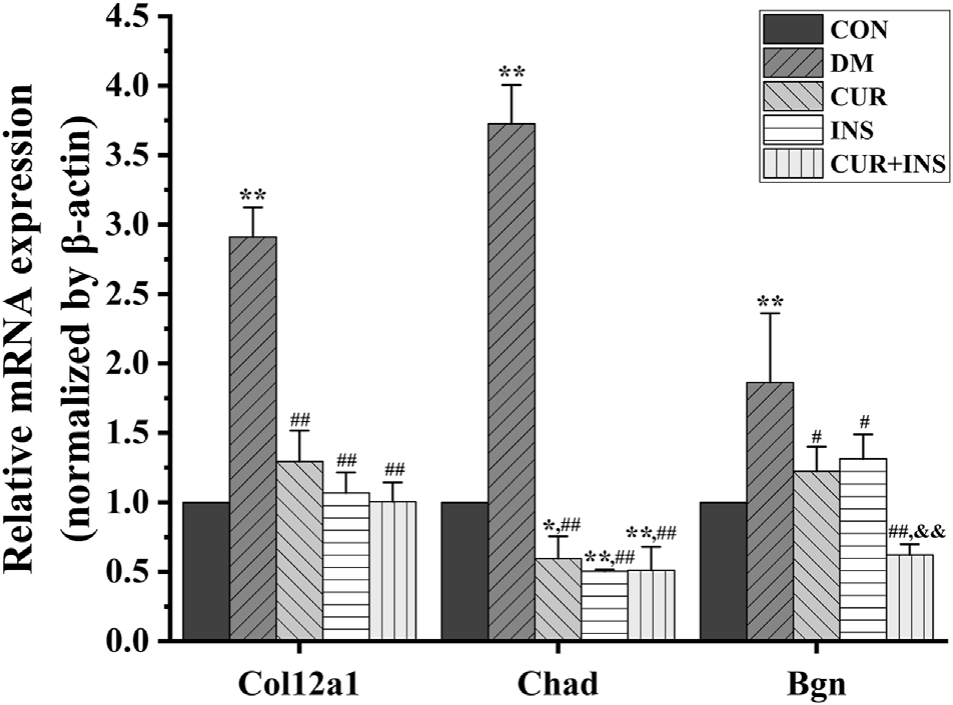
Real-time qPCR analysis for main DEGs expression. CON, control group; DM, STZ-induced diabetic model group; CUR, 200 mg/kg curcumin treatment group; INS, long-acting insulin treatment group; CUR + INS, 200 mg/kg curcumin and long-acting insulin treatment group. All data are expressed as the mean ± standard deviation (*n* > 3). ^**^ *p* < 0.01 vs. the CON group; ^*^ *p* < 0.05 vs. the CON group; ^##^ *p* < 0.01 vs. the DM group; ^#^ *p* < 0.05 vs. the DM group; ^&&^ *p* < 0.01vs. the CUR group.

KEGG pathway analysis was used to identify further the most critical biochemical metabolic and signal transduction pathway related to the therapeutic mechanism of curcumin. According to KEGG enrichment results (Figure 6), the significantly differentially expressed genes between the DM group and CON group were mainly enriched in insulin secretion, isoquinoline alkaloid biosynthesis, and phenylalanine metabolism (Figure 6A). Notably, among the three comparisons (CUR vs. DM, INS vs. DM, and (CUR + INS) vs. DM), we found that the major significant enrichment KEGG pathways all involved ECM-receptor interaction, proteoglycans, focal adhesion, and glycosaminoglycan binding proteins (Figures 6B–D). Meanwhile, we confirmed the expression level of main differential genes with connection to the above major significant enrichment GO terms and KEGG pathways using qRT-PCR (Figure 7). The results of qRT-PCR experiments for these genes were consistent with the transcriptome profiling.

## Discussion

Diabetic retinopathy (DR), a severe neurovascular complication of diabetes, is the primary cause of visual impairment and blindness in the working-age population (Whitehead et al., 2018). The failure of pancreatic beta cells to produce insulin, and the impairment of insulin action, play a central role in the disruption of glycemic homeostasis, leading to hyperglycemia, a hallmark of DM (Thomas and Philipson., 2015). A cascade of reactions triggered by elevated blood glucose can lead to the destruction of the blood barrier and the development of neovascularization and macular edema in the retina, ultimately threatening sight (Heng et al., 2013). In order to prevent the development of chronic diabetes complications, including diabetic retinopathy, some patients need to use insulin to optimize blood glucose control. Unfortunately, side effects, including acute hypoglycemia and insulin resistance, are frequently observed in patients with long-term injected insulin treatment, which has driven research into adjunctive agent use in insulin therapy. Recently, studies have proposed and confirmed that natural polyphenol compounds such as insulin sensitizers and adjuvant therapy drugs, can improve glycemic control in insulin-treated T1D-like rats (Yonamine et al., 2016). Curcumin is a natural, polyphenolic antioxidant extracted from the root of *Curcuma longa*, which is widely used in diabetes and diabetic cardiovascular complications (Li et al., 2020). It has also been considered as a potential therapeutic agent in the treatment of several eye disorders (Radomska-Leśniewska et al., 2019). In recent years, accumulating evidence has demonstrated that orally administered curcumin can prevent weight loss, reduce blood hemoglobin, glucose, and glycosylated hemoglobin levels, and improve insulin sensitivity in diabetic rat models (Nabavi et al., 2015b). In our study, the elevated blood glucose induced by STZ injection was reversed by curcumin or insulin, or a combination of both. By comparing the hypoglycemic efficacy of different groups, we found that curcumin was not as stable as insulin in controlling blood glucose. In order to highlight the feature of DR, we used STZ to establish a model similar to the pathogenesis of human type 1 diabetes, which made serious destruction of islet function. Consequently, blood glucose is difficult to control. Although the results indicated curcumin had an insignificant effect on improving weight loss in diabetic rats, curcumin has a strong synergistic effect that enhances the stability of insulin glycemic control and anti-apoptotic ability, as well as reducing polydipsia and polyuria. In a high glucose environment, our staining results showed that edema and apoptosis of the retina were markedly increased. After the intervention, retinal morphological damage was significantly alleviated in diabetic rats. It was worth noting that curcumin had a better effect on reducing photoreceptor apoptosis than insulin. Photoreceptors are known to have the highest density of mitochondria in the outer retina (Winkler et al., 2003), which makes them more vulnerable to oxidative stress induced by local high-glucose concentrations (Maisto et al., 2017). The molecular structure of curcumin contains two important radical scavenging groups, a CH_2_ group of the β-diketone moiety and an ortho-methoxy phenolic group, both of which can provide hydrogen to bind with oxygen radicals (Kocaadam and Şanlier, 2017), which protected photoreceptor from oxidative stress induced by high glucose. Autophagy, as a crucial and evolutionarily conserved mechanism that regulates the steady-state function of cells, could inhibit the activation of apoptosis by suppressing the stimulation of apoptosis-related caspase (Young et al., 2012). The complex interplay between autophagy and apoptosis plays an important role in determining the degree of apoptosis and DR promotion (Rosa et al., 2016). In this study hyperglycemia inhibited retinal autophagy instigated LC3B-Ⅱ and p62 downregulation. However, insulin treatment significantly increased LC3B-Ⅱ expression and maintained p62 expression. Therefore, we speculated that enhancing autophagy activity was one of the potential mechanisms of insulin therapy for diabetic retinopathy.

In contrast to the Ins group, the combination therapy of curcumin and insulin reduced p62 and LC3B-Ⅱ protein expression. Recently, several clinical studies have confirmed that curcumin can significantly improve insulin resistance in diabetic patients and has insulin-sensitizing effects (Ghorbani et al., 2014; Thota et al., 2019; Heshmati et al., 2021). However, sometimes higher insulin is not necessarily better, and chronic exposure to high ambient insulin concentrations results in unbalanced insulin anabolic activity and suppressed autophagy (Kolb et al., 2020). Therefore, we speculated that the addition of curcumin increased the sensitivity of cells to insulin and achieved the effect of high insulin concentration at an otherwise appropriate dose. This inhibited autophagy, as evidenced by the decreased levels of p62 and LC3B-Ⅱ proteins in the retina of rats in the (CUR + INS) group. Tu et al. revealed that curcumin induced renal autophagy by downregulating the expression of p62, PI3K, p-AKT, and p-mTOR proteins, and upregulating LC3B-Ⅱ, Nrf2, and HO-1 levels (Di Tu et al., 2020). However, our results suggested that curcumin increased LC3B-Ⅱ expression and p62 also increased. The accumulation of p62 was related to the impaired binding of autophagosomes and lysosomes, or the delay in autolysosome degradation. Therefore, whether the effect of curcumin on retinal autophagy is the same as previously described needs to be verified by further experiments.

Although many factors such as autophagy, advanced glycation end products (AGEs), inflammation, and interaction between these factors have been considered to be involved in diabetic retinopathy pathogenesis (Semeraro et al., 2015; Lopes de Faria et al., 2016; Sahajpal et al., 2019), persistent hyperglycemia-mediated overproduction of reactive oxygen species is a primary pathological mechanism in DR (Whitmire et al., 2011; Behl et al., 2016). Nuclear factor-E2 related factor 2 (Nrf2) is an important sensor protein of xenobiotic toxic substances and oxidants. Recent research has shown that Nrf2 activation and upregulation could dramatically alleviate diabetes-induced retinal oxidation (Liu et al., 2017; Dong et al., 2019; Li et al., 2019). Furthermore, Nrf2 has been identified as a novel therapeutic strategy in diabetic retinopathy (Nabavi et al., 2016). Curcumin has been demonstrated to exert antioxidative and anti-inflammatory properties through activation of the Nrf2 pathway in diabetes-associated myocardial fibrosis and renal injury (Yang et al., 2015; Xiang et al., 2020). Thus, the potential role of Nrf2 under high-glycemic-induced retinal damage combined with curcumin treatment deserves investigation. In this study, oxidative stress was enhanced in the retina of diabetic rats, demonstrated by an increased MDA level, higher GSSG/GSH ratio, and reduced SOD and T-GSH levels. The increased ROS level induced the adaptive activation of the Nrf2-HO-1 pathway to counteract REDOX imbalance in the retina, which significantly raised Nrf2 and HO-1 expression levels and the number of Nrf2 nuclear-stained cells. After curcumin intervention, the MDA level, the HO-1 expression level of Nrf2 downstream, and the number of Nrf2 nuclear-stained cells were significantly decreased in the retina of diabetic rats. On the one hand, curcumin alleviated the compensatory activation of the Nrf2 pathway induced by oxidative stress, by virtue of its antioxidant ability to transfer hydrogen atoms to free radicals (Kocaadam and Şanlier, 2017). On the other hand, curcumin did not induce Nrf2 activation by promoting nuclear translocation of Nrf2 as previously reported in other complications of diabetes (Xiang et al., 2020). The disparities in curcumin effect in the different tissue of diabetic rats may be ascribed to different durations of diabetes or perhaps the different blood concentrations of the curcumin. However, despite receiving insulin to enhance glycemic control, the Nrf2 pathway in diabetic retinas remained in an over-activated state. This phenomenon of persistent aberrant expression of antioxidant genes, regardless of glucose normalization, is known as “metabolic memory”; a serious dysfunction observed in DR. Despite diabetes patients undergoing intensive glycemic control, there would be some reprogramming in self-supported oxidative stress, chronic inflammation, and epigenetic modification during the early phases of diabetes mellitus, leading to irreversible changes in gene expression and mitochondrial function (Testa et al., 2017). Therefore, in order to minimize the impact of hyperglycemia on tissues, in addition to intensive and rapid glycemic control treatment in diabetic individuals, the novel combined therapeutic approaches have been proposed. These include the use of natural bioactive compounds capable of inhibiting the biochemical cascades triggered by advanced glycation, reactive oxygen species (ROS), and inflammation, thus effectively retarding DR development (Lima et al., 2020). Our results found that the combination of insulin and curcumin significantly reduced MDA level as well as restored Nrf2 pathway activity to the normal level, which indicated that the combination of curcumin and insulin not only enhanced glycemic control but also reduced the damage caused by metabolic memory to retinal antioxidant genes.

A transcriptome is a complete set of gene transcripts, or RNA species, transcribed in cells or tissues for specific physiological or pathological conditions. It comprises both coding RNA translated into proteins, and non-coding RNA involved in post-transcriptional control, which further influences gene expression. The purpose of transcriptome research is to evaluate the variation of gene transcript abundance across different experimental conditions through differential expression analysis and then identify coregulatory genes through cluster analysis to ascertain the biological mechanism and pathway (Chambers et al., 2019). In this study, transcriptome analysis demonstrated that supplementation with curcumin either alone, or when combined with insulin, mainly enriched the genes that participate in the maintenance of retinal microenvironmental homeostasis, including ECM-receptor interaction, proteoglycans, and the AGE-RAGE signaling pathway. Compared with the CON group, the expression levels of genes involved in ECM receptor interactions and the AGE-RAGE signaling pathway, including *Col12a1*, *Chad*, *Bgn*, etc., were significantly increased in the DM group. Advanced glycation end products (AGEs) are the products of oxidative stress induced glycation reactions (Vistoli et al., 2013). The accumulation of AGEs induced by high glucose could prevent tissue integrity and the maintenance of normal functions by modifying the long-life structural components of the basement membrane or extracellular matrix (ECM). This disrupts normal cell-matrix contact or prevents physiologic cellular growth and intercellular contact (Bohlender et al., 2005). After treatment with curcumin or insulin or a combination of both, the elevated expression levels of genes were reversed, including *Col12a1*, *Chad*, *Bgn*, etc. The inhibition of the AGEs-RAGE signaling pathway in each drug intervention group led to the alleviation of oxidative stress, which is consistent with our molecular biological detection results. Furthermore, we found that the suppression effect of curcumin was no less effective than insulin. Alizadeh et al. demonstrated that curcumin plays a beneficial role in different age-related chronic diseases by inhibiting AGEs formation and AGEs-induced disturbances (Alizadeh and Kheirouri, 2017). Therefore, we proposed that curcumin could protect the integrity of the retinal structure by inhibiting the activity of the AGE-RAGE signaling pathway. This may be a new therapeutic target for curcumin in diabetic retinopathy.

There were several limitations to this study. Firstly, the experiment failed to observe the effect of curcumin on improving weight loss due to the type 1 diabetic rat model, and the new experimental animal models need to be applied. Secondly, the small sample size of each group in the transcriptome analysis led to the increase of sampling error. The occurrence of outliers would directly affect the differential expression gene pairwise comparison results between our groups. In future experiments, we would further expand the sample size to reduce the influence of outliers so as to screen out the main targets of curcumin more accurately. Finally, in this study, the effect of curcumin on promoting Nrf2 nuclear translocation in the retina of diabetic rats was not observed, so it should be considered to prolong the course of diabetes in future experiments and change the administration mode of curcumin to improve its drug concentration in retinal blood vessels. Furthermore, there was a suggestion for the future research of curcumin in ophthalmology. In our study, we found that curcumin has a significant advantage in improving retinal photoreceptor apoptosis in diabetic rats. Combined with the characteristics of high mitochondrial density, high oxygen consumption, and long-term exposure to light of photoreceptors, we suggest further exploring the protective mechanism of curcumin on retinal photoreceptors under hyperglycemia or light irradiation.

## Conclusion

In summary, our data indicated that treatment with curcumin, insulin, or combination therapy had an essential role in regulating the blood glucose, reducing oxidative stress, and improved histopathological damage in diabetic rats. Curcumin not only significantly reduced retinal edema, but also had better anti-photoreceptor apoptosis effect than insulin. The anti-photoreceptor apoptosis effect of insulin was related to its potent activities in correcting the imbalance between diabetic retinal autophagy and apoptosis. Curcumin has a strong synergistic effect that enhanced the stability of insulin glycemic control and anti-apoptotic ability as well as maintain the homeostasis of Nrf2 endogenous antioxidant pathway in the early stages of diabetes. Transcriptomic analyses revealed that curcumin either alone, or combined with insulin inhibited the AGE-RAGE signaling pathway and the extracellular matrix (ECM)-receptor interaction. This served as a new therapeutic target for curcumin in diabetic retinopathy. Thus, at the early stage of diabetes, curcumin can be used to alleviates diabetic retinal injury through its anti-oxidative effect. If taking curcumin as a potential complementary therapeutic option in combination with antihyperglycemic agents, which would lead to more effective therapeutic outcomes against diabetic complications.

## Data Availability

The datasets presented in this study can be found in online repositories. The names of the repository/repositories and accession number(s) can be found below: https://www.ncbi.nlm.nih.gov/sra/PRJNA769974.
