# Supplementary material for: Curcumin is a Potential Adjuvant to Alleviates Diabetic Retinal Injury via Reducing Oxidative Stress and Maintaining Nrf2 Pathway Homeostasis
Source: Front Pharmacol. 2021 Dec 10;12:796565. doi: 10.3389/fphar.2021.796565 (PMC8702852; doi:10.3389/fphar.2021.796565)
Supplement: Supplementary file 1 [file DataSheet1.docx]

Supplementary Material

# Supplementary Tables

**Table S1. Primers used for (q)PCR and sequencing**

| Gene (Rat) | Forward primer (5’ to 3’) | Reverse primer (5’ to 3’) | | Size (bp) |
| --- | --- | --- | --- | --- |
| Nrf2 | CGATTAGAGGCTCATCTCACAA | GTTGAATTGCTCCTTGGACATC | | 129 |
| p62 | GCACTACCGCGATGAGGATG | TCACAAATCACGTTGGGGTG | | 189 |
| HO-1 | CTAAGACCGCCTTCCTGCTC | GCGGTGTCTGGGATGAACTA | | 195 |
| LC3-Ⅱ | TTGGTCAAGATCATCCGGCG | AGCCGAAGGTTTCTTGGGAG | | 175 |
| β-actin | CGCGAGTACAACCTTCTTGC | CCTTCTGACCCATACCCACC | | 211 |
| Col12a1 | GTGTGGCTGATGTGGACTAC | CCAGATAGACGAGAGGACAACT | | 165 |
| Chad | ACCAATTGCCCTCCACCTTC | GCAGGTAGCATCTGGTCGAG | | 137 |
| Bgn | TTGGAGGATCTACTTCGCTACT | AGATAGACAACCTGGAGGAGTT | | 185 |
| Col1a1 | CGAGTATGGAAGCGAAGGTT | CTTGAGGTTGCCAGTCTGTT | | 148 |
| NEWGENE_621351 | GGCAACAGCAGATTCACCTAC | GCAGGCGAGATGGCTTATTC | 109 | |

**Table S2. List of differentially expressed genes in the DM group vs. CON group**

| Gene ID | Gene name | Fold change | P-Value | Level |
| --- | --- | --- | --- | --- |
| 61358 | AC129365.1 | -2.625768 | 1.65E-09 | Decreased |
| 40350 | Mir675 | 1.54241663 | 1.68E-07 | Increased |
| 49882 | Adcyap1 | -1.1453263 | 2.13E-07 | Decreased |
| 03336 | Mybph | 2.15676195 | 3.38E-07 | Increased |
| 01399 | Tbx5 | -3.2208214 | 1.03E-06 | Decreased |
| 57989 | Zp2 | 2.75990432 | 1.07E-06 | Increased |
| 11591 | Mettl21c | 2.98352491 | 1.61E-06 | Increased |
| 04327 | Ddc | 1.30744786 | 3.43E-06 | Increased |
| 07041 | Abcg2 | 1.53101799 | 4.85E-06 | Increased |
| 47295 | Prr22 | 1.26985233 | 5.25E-06 | Increased |
| 25691 | Pla2g7 | 1.80053445 | 5.84E-06 | Increased |
| 40108 | RGD1565355 | 1.43184091 | 6.65E-06 | Increased |
| 50146 | LOC100909732 | 1.231538 | 1.06E-05 | Increased |
| 20308 | Ech1 | 1.28950097 | 1.63E-05 | Increased |
| 19321 | Cck | -1.3042728 | 1.63E-05 | Decreased |
| 52646 | AABR07029836.1 | 1.93333713 | 2.7E-05 | Increased |
| 09047 | Sln | 1.59919309 | 4.58E-05 | Increased |

**Table S3. List of differentially expressed genes in the CUR group vs. DM group**

| Gene ID | Gene name | Fold change | *P*-Value | Level |
| --- | --- | --- | --- | --- |
| 16700 | Tcf21 | -8.51679 | 1.33E-09 | Decreased |
| 45729 | AC117058.1 | 5.90693 | 1.48E-08 | Increased |
| 24082 | Gldn | -1.10771 | 3.77E-06 | Decreased |
| 15902 | Cpxm2 | -1.63923 | 4.77E-06 | Decreased |
| 11841 | Map2 | -1.32283 | 1.14E-05 | Decreased |
| 43098 | Mt2A | 1.302311 | 1.24E-05 | Increased |
| 61152 | AABR07018038.3 | -4.94958 | 1.37E-05 | Decreased |
| 28108 | Cytl1 | 1.992189 | 2.62E-05 | Increased |
| 58470 | Col12a1 | -2.5356 | 3.00E-05 | Decreased |
| 03304 | Chad | -2.30142 | 3.11E-05 | Decreased |

**Table S4. List of differentially expressed genes in the INS group vs. DM group**

| Gene ID | Gene name | Fold change | *P*-Value | Level |
| --- | --- | --- | --- | --- |
| 15902 | Cpxm2 | -2.48315 | 7.68E-19 | Decreased |
| 29911 | Cilp | -2.66641 | 1.11E-14 | Decreased |
| 58470 | Col12a1 | -3.52093 | 4.18E-13 | Decreased |
| 15076 | Cyp26b1 | -2.67228 | 1.16E-11 | Decreased |
| 16945 | Pla2g2a | -2.72512 | 3.73E-11 | Decreased |
| 51399 | Col10a1 | -3.9401 | 1.22E-10 | Decreased |
| 51399 | Col10a1 | -3.9401 | 1.22E-10 | Decreased |
| 11101 | Twist1 | -2.09347 | 2.69E-10 | Decreased |
| 10529 | Thbs2 | -2.44727 | 2.72E-10 | Decreased |
| 55962 | Bgn | -2.24585 | 6.75E-10 | Decreased |
| 10259 | Esrrb | -1.27567 | 8.86E-10 | Decreased |
| 48472 | Comp | -4.16067 | 1.23E-09 | Decreased |
| 03183 | Fmod | -2.39566 | 3.69E-09 | Decreased |
| 26497 | Pigc | -1.13207 | 3.75E-09 | Decreased |
| 20622 | Cilp2 | -3.24201 | 4.26E-09 | Decreased |
| 13720 | Aebp1 | -1.16502 | 5.03E-09 | Decreased |
| 21220 | Cpxm1 | -1.55751 | 1.01E-08 | Decreased |
| 14443 | Pde5a | -1.33355 | 4.18E-08 | Decreased |
| 19138 | Clec11a | -2.14217 | 4.23E-08 | Decreased |
| 45829 | Thbs1 | -1.69525 | 1.41E-07 | Decreased |
| 47295 | Prr22 | -1.37593 | 2.02E-07 | Decreased |
| 26059 | Paqr6 | -1.09555 | 2.21E-07 | Decreased |
| 05825 | Lyz2 | -1.22313 | 3.56E-07 | Decreased |
| 05825 | Lyz2 | -1.22313 | 3.56E-07 | Decreased |
| 19352 | Emc6 | 1.054374 | 3.74E-07 | Increased |
| 08336 | Tnfrsf11b | -1.78073 | 4.84E-07 | Decreased |
| 13269 | Tnfsf10 | -1.35078 | 5.37E-07 | Decreased |
| 32708 | RT1-Bb | -3.19065 | 9.77E-07 | Decreased |
| 51179 | Vps25 | 3.147653 | 1.01E-06 | Increased |
| 25670 | Shisa3 | -2.9308 | 1.01E-06 | Decreased |
| 31475 | Col16a1 | -1.50978 | 1.03E-06 | Decreased |
| 19244 | Mxra8 | -1.20086 | 1.25E-06 | Decreased |
| 10977 | Igfbp6 | -1.48219 | 1.37E-06 | Decreased |
| 52925 | NEWGENE_621351 | -2.10942 | 1.88E-06 | Decreased |
| 10840 | Adamtsl3 | -1.81451 | 2.21E-06 | Decreased |
| 03897 | Col1a1 | -1.80556 | 3.03E-06 | Decreased |
| 04516 | Itgbl1 | -2.01799 | 3.89E-06 | Decreased |
| 22490 | LOC100361008 | 1.577033 | 5.22E-06 | Increased |
| 11292 | NEWGENE_621351 | -1.9698 | 7.01E-06 | Decreased |
| 20679 | Icam1 | -1.02806 | 1.1E-05 | Decreased |
| 11841 | Map2 | -1.10174 | 1.56E-05 | Decreased |
| 09594 | Snai1 | -2.05285 | 1.68E-05 | Decreased |
| 08947 | Cpz | -1.55108 | 1.97E-05 | Decreased |
| 08947 | Cpz | -1.55108 | 1.97E-05 | Decreased |
| 01627 | Abi3bp | -1.64409 | 2.39E-05 | Decreased |
| 43451 | Spp1 | -2.14718 | 2.74E-05 | Decreased |
| 19211 | Olfml3 | -1.27271 | 3.1E-05 | Decreased |
| 10253 | Cd163 | -1.66276 | 3.13E-05 | Decreased |
| 03947 | Ntn1 | -1.74275 | 3.14E-05 | Decreased |
| 03304 | Chad | -3.86096 | 3.16E-05 | Decreased |
| 15461 | Serpine2 | -1.57436 | 4.08E-05 | Decreased |
| 02041 | Boc | -1.42661 | 4.68E-05 | Decreased |
| 48043 | F2r | -1.04343 | 5.75E-05 | Decreased |
| 03620 | Fmo3 | -2.88539 | 5.94E-05 | Decreased |
| 46254 | Adgre1 | -1.01925 | 6.57E-05 | Decreased |
| 49056 | AABR07051450.1 | -1.28079 | 6.84E-05 | Decreased |
| 06320 | Ptges | -1.44409 | 7.43E-05 | Decreased |
| 00451 | RT1-Ba | -1.91159 | 7.89E-05 | Decreased |
| 45992 | Tlr8 | -1.47915 | 7.98E-05 | Decreased |
| 11360 | Dkk2 | -1.62974 | 8.76E-05 | Decreased |
| 27808 | Lilra5 | -2.18518 | 9.45E-05 | Decreased |
| 09589 | Angptl7 | -3.86817 | 9.77E-05 | Decreased |
| 10832 | Pdgfrl | -1.78645 | 0.000101 | Decreased |
| 00700 | Tmem119 | -1.52523 | 0.000102 | Decreased |
| 05998 | Smoc1 | -1.30486 | 0.000117 | Decreased |
| 62101 | Ace | -1.43088 | 0.000127 | Decreased |
| 06741 | Podnl1 | -2.69225 | 0.000127 | Decreased |
| 15498 | Il17rb | 2.03139 | 0.00013 | Increased |
| 25001 | Pcolce | -1.42395 | 0.000148 | Decreased |
| 22565 | Lrrc25 | -1.24822 | 0.000156 | Decreased |
| 04610 | Lum | -1.3272 | 0.000171 | Decreased |
| 49422 | LOC108348047 | -1.45963 | 0.000188 | Decreased |
| 49422 | LOC108348047 | -1.45963 | 0.000188 | Decreased |
| 49422 | LOC108348047 | -1.45963 | 0.000188 | Decreased |
| 49422 | LOC108348047 | -1.45963 | 0.000188 | Decreased |
| 17676 | Plvap | -1.28899 | 0.000188 | Decreased |
| 01254 | Col6a2 | -1.2341 | 0.000213 | Decreased |
| 07202 | Sema3d | -1.22063 | 0.00022 | Decreased |
| 14361 | Edn1 | -1.28298 | 0.000226 | Decreased |
| 16366 | Colec12 | -1.32395 | 0.000231 | Decreased |
| 16826 | Pla2g2d | -2.07642 | 0.000235 | Decreased |
| 04699 | Fibin | -1.15837 | 0.000237 | Decreased |
| 09867 | Tgfb3 | -1.11493 | 0.000238 | Decreased |
| 21243 | Siglec1 | -1.58277 | 0.000244 | Decreased |
| 12619 | Epor | -2.06672 | 0.000247 | Decreased |
| 10617 | Scube1 | -1.32087 | 0.000285 | Decreased |
| 17987 | Ucma | -3.93867 | 0.000289 | Decreased |
| 14465 | Oca2 | -1.29978 | 0.000307 | Decreased |
| 05931 | Cpq | -1.00299 | 0.000313 | Decreased |

**Table S5. List of differentially expressed genes in the (CUR+INS) group vs. DM group**

| Gene ID | Gene name | Fold change | *P*-Value | Level |
| --- | --- | --- | --- | --- |
| 18735 | Cd74 | -1.75952 | 6.54E-48 | Decreased |
| 25670 | Shisa3 | -3.95948 | 1.29E-33 | Decreased |
| 13720 | Aebp1 | -1.94025 | 2.11E-31 | Decreased |
| 03304 | Chad | -4.81112 | 2.24E-31 | Decreased |
| 32844 | RT1-Da | -2.0873 | 3.03E-27 | Decreased |
| 14288 | Fn1 | -2.00073 | 2.07E-24 | Decreased |
| 15902 | Cpxm2 | -2.9716 | 2.75E-24 | Decreased |
| 14443 | Pde5a | -2.04227 | 3.45E-21 | Decreased |
| 15076 | Cyp26b1 | -3.6015 | 7.79E-21 | Decreased |
| 09589 | Angptl7 | -5.08735 | 9.56E-21 | Decreased |
| 28627 | Hmcn1 | -2.46834 | 1.49E-20 | Decreased |
| 10666 | Ccn5 | -4.84889 | 4.35E-20 | Decreased |
| 20622 | Cilp2 | -4.47812 | 1.21E-19 | Decreased |
| 58470 | Col12a1 | -4.43312 | 1.35E-19 | Decreased |
| 31665 | Ace2 | -2.20286 | 2.93E-19 | Decreased |
| 05998 | Smoc1 | -2.53806 | 3.76E-18 | Decreased |
| 05854 | Angpt1 | -2.85216 | 3.94E-18 | Decreased |
| 21084 | AABR07006310.1 | -2.25218 | 1.19E-17 | Decreased |
| 55962 | Bgn | -2.63498 | 4.16E-17 | Decreased |
| 21437 | AABR07073181.1 | -2.40445 | 7.62E-17 | Decreased |
| 18434 | Stab1 | -2.75528 | 2.90E-16 | Decreased |
| 61910 | Igfbp3 | -1.74805 | 6.88E-16 | Decreased |
| 18903 | Pik3r1 | -1.33572 | 1.77E-15 | Decreased |
| 02171 | Phldb2 | -2.14855 | 5.48E-15 | Decreased |
| 05964 | Nr4a3 | -2.65802 | 1.02E-14 | Decreased |
| 03897 | Col1a1 | -2.44627 | 1.08E-14 | Decreased |
| 31475 | Col16a1 | -2.1443 | 4.20E-14 | Decreased |
| 62125 | Aox3 | -1.84817 | 6.95E-14 | Decreased |
| 05695 | Mgp | -2.14182 | 7.78E-14 | Decreased |
| 08336 | Tnfrsf11b | -2.18055 | 9.69E-14 | Decreased |
| 11292 | NEWGENE_621351 | -2.60751 | 1.08E-13 | Decreased |
| 25625 | Rnase4 | -1.25574 | 1.30E-13 | Decreased |
| 26497 | Pigc | -1.55043 | 1.33E-13 | Decreased |
| 06741 | Podnl1 | -9.97587 | 1.96E-13 | Decreased |
| 52925 | NEWGENE_621351 | -2.45028 | 3.24E-13 | Decreased |
| 19138 | Clec11a | -2.41462 | 3.28E-13 | Decreased |
| 17676 | Plvap | -1.44733 | 9.74E-13 | Decreased |
| 16752 | Crispld2 | -1.39445 | 1.01E-12 | Decreased |
| 16627 | Eya4 | -3.12731 | 2.04E-12 | Decreased |
| 17307 | Prss23 | -1.625 | 2.57E-12 | Decreased |
| 16678 | Angptl2 | -1.6185 | 4.49E-12 | Decreased |
| 15283 | Nt5c1a | -3.06491 | 5.52E-12 | Decreased |
| 49124 | Mylk4 | -4.0862 | 7.75E-12 | Decreased |
| 02041 | Boc | -2.40212 | 1.22E-11 | Decreased |
| 46683 | Lilrb3 | -3.906 | 1.43E-11 | Decreased |
| 21201 | Txnip | -1.32921 | 1.58E-11 | Decreased |
| 16480 | Acbd7 | 1.283233 | 3.58E-11 | Increased |
| 59326 | Abca9 | -1.54996 | 3.82E-11 | Decreased |
| 59326 | Abca9 | -1.54996 | 3.82E-11 | Decreased |
| 33496 | Igdcc4 | -1.45256 | 4.68E-11 | Decreased |
| 01627 | Abi3bp | -2.22719 | 5.43E-11 | Decreased |
| 14872 | Sec24d | -1.62992 | 6.14E-11 | Decreased |
| 00700 | Tmem119 | -2.11859 | 8.02E-11 | Decreased |
| 03120 | Prelp | -1.54431 | 1.44E-10 | Decreased |
| 01515 | Map3k20 | -2.15956 | 1.90E-10 | Decreased |
| 55936 | Trnp1 | 1.037102 | 3.54E-10 | Increased |
| 19244 | Mxra8 | -1.92197 | 4.51E-10 | Decreased |
| 01249 | Col6a1 | -2.21006 | 4.69E-10 | Decreased |
| 15461 | Serpine2 | -2.31001 | 5.06E-10 | Decreased |
| 15354 | Aox1 | -1.83099 | 6.64E-10 | Decreased |
| 16058 | Kazald1 | -2.43954 | 6.96E-10 | Decreased |
| 03736 | Col5a2 | -2.08346 | 8.66E-10 | Decreased |
| 03620 | Fmo3 | -2.595 | 9.13E-10 | Decreased |
| 54360 | Tspan11 | -2.18129 | 9.35E-10 | Decreased |
| 24101 | Phkb | -1.86879 | 9.66E-10 | Decreased |
| 21840 | Cped1 | -2.56904 | 1.07E-09 | Decreased |
| 29911 | Cilp | -3.06022 | 1.13E-09 | Decreased |
| 09594 | Snai1 | -2.54118 | 1.23E-09 | Decreased |
| 01656 | Kcnj15 | -3.4068 | 1.28E-09 | Decreased |
| 00373 | AABR07044900.1 | -3.99365 | 1.33E-09 | Decreased |
| 21261 | Rassf2 | -1.73547 | 1.41E-09 | Decreased |
| 00288 | Scarf2 | -1.4016 | 1.66E-09 | Decreased |
| 51440 | Ppp1r12b | -1.02845 | 1.75E-09 | Decreased |
| 16700 | Tcf21 | -8.40033 | 2.18E-09 | Decreased |
| 27489 | Mn1 | -1.57084 | 2.19E-09 | Decreased |
| 09431 | Tbc1d4 | -1.60537 | 2.24E-09 | Decreased |
| 10840 | Adamtsl3 | -2.20557 | 2.39E-09 | Decreased |
| 10183 | Gask1b | -1.88873 | 2.66E-09 | Decreased |
| 01414 | Serpine1 | -3.36551 | 2.76E-09 | Decreased |
| 36960 | Abcc9 | -1.77716 | 3.36E-09 | Decreased |
| 25001 | Pcolce | -2.27325 | 3.38E-09 | Decreased |
| 15488 | Tead1 | -1.08324 | 3.43E-09 | Decreased |
| 15024 | Mcoln3 | -3.90589 | 3.61E-09 | Decreased |
| 00081 | Antxr2 | -1.98798 | 4.38E-09 | Decreased |
| 00869 | Arhgef6 | -1.79088 | 4.88E-09 | Decreased |
| 16866 | Fhl2 | -2.04145 | 4.89E-09 | Decreased |
| 09565 | Pdk4 | -3.61601 | 5.50E-09 | Decreased |
| 22565 | Lrrc25 | -2.12156 | 6.25E-09 | Decreased |
| 23400 | Dtx3l | -1.43957 | 6.82E-09 | Decreased |
| 11101 | Twist1 | -2.07359 | 6.92E-09 | Decreased |
| 42889 | Alpg | -2.16589 | 7.85E-09 | Decreased |
| 28910 | Ccdc9b | -1.24178 | 7.95E-09 | Decreased |
| 03720 | Prrx1 | -2.47676 | 1.03E-08 | Decreased |
| 00824 | Dse | -1.79303 | 1.03E-08 | Decreased |
| 15036 | Ccn2 | -1.88208 | 1.22E-08 | Decreased |
| 12804 | C1qc | -1.58788 | 1.31E-08 | Decreased |
| 12804 | C1qc | -1.58788 | 1.31E-08 | Decreased |
| 04516 | Itgbl1 | -2.70277 | 1.37E-08 | Decreased |
| 20652 | Tgfb1 | -1.01478 | 1.42E-08 | Decreased |
| 15071 | Zim1 | -2.78562 | 1.46E-08 | Decreased |
| 34190 | Ighm | -2.63115 | 1.50E-08 | Decreased |
| 48924 | Islr | -2.05678 | 1.65E-08 | Decreased |
| 03183 | Fmod | -2.72384 | 1.75E-08 | Decreased |
| 12749 | C1qb | -1.30759 | 1.80E-08 | Decreased |
| 12830 | Paqr8 | -1.17395 | 1.85E-08 | Decreased |
| 58329 | Prrx2 | -1.69852 | 2.05E-08 | Decreased |
| 29938 | Pik3c2b | -1.42309 | 2.15E-08 | Decreased |
| 00008 | Alx4 | -3.24474 | 2.73E-08 | Decreased |
| 13265 | Tgfbr2 | -2.00593 | 2.77E-08 | Decreased |
| 54508 | Foxp2 | -1.28437 | 2.77E-08 | Decreased |
| 21220 | Cpxm1 | -2.25369 | 3.47E-08 | Decreased |
| 19181 | Synpo | -1.56083 | 3.63E-08 | Decreased |
| 02134 | Gbp6 | -2.26526 | 3.99E-08 | Decreased |
| 25502 | Arhgef37 | -1.83985 | 4.49E-08 | Decreased |
| 49491 | RT1-DMb | -1.55062 | 4.75E-08 | Decreased |
| 57522 | LOC103689968 | -1.58494 | 4.91E-08 | Decreased |
| 14776 | Adcy7 | -1.80118 | 5.75E-08 | Decreased |
| 30210 | Fndc1 | -1.64934 | 5.84E-08 | Decreased |
| 03510 | Fmo2 | -2.55483 | 6.17E-08 | Decreased |
| 10832 | Pdgfrl | -2.63527 | 6.45E-08 | Decreased |
| 10529 | Thbs2 | -3.10284 | 7.38E-08 | Decreased |
| 16945 | Pla2g2a | -2.45432 | 9.97E-08 | Decreased |
| 14182 | Tns1 | -1.66576 | 1.05E-07 | Decreased |
| 08947 | Cpz | -1.67549 | 1.25E-07 | Decreased |
| 08947 | Cpz | -1.67549 | 1.25E-07 | Decreased |
| 04936 | Sdc2 | -1.30986 | 1.31E-07 | Decreased |
| 23208 | Myorg | -1.92866 | 1.45E-07 | Decreased |
| 15086 | Plin1 | -4.055 | 1.46E-07 | Decreased |
| 09385 | Pik3cg | -1.49927 | 1.54E-07 | Decreased |
| 13269 | Tnfsf10 | -1.71599 | 1.73E-07 | Decreased |
| 05825 | Lyz2 | -1.74424 | 1.82E-07 | Decreased |
| 05825 | Lyz2 | -1.74424 | 1.82E-07 | Decreased |
| 53550 | Itga1 | -1.5273 | 1.82E-07 | Decreased |
| 04812 | Sema6d | -1.50637 | 1.91E-07 | Decreased |
| 20716 | Axl | -1.41694 | 1.94E-07 | Decreased |
| 01254 | Col6a2 | -1.89486 | 2.27E-07 | Decreased |
| 01821 | Adipoq | -4.00547 | 2.43E-07 | Decreased |
| 33338 | Gimap6 | -1.42057 | 2.57E-07 | Decreased |
| 02369 | Rgs8 | 1.453844 | 2.57E-07 | Increased |
| 61484 | Adamts2 | -2.11919 | 2.58E-07 | Decreased |
| 30689 | Ms4a6bl | -2.07912 | 2.61E-07 | Decreased |
| 11360 | Dkk2 | -2.0221 | 2.66E-07 | Decreased |
| 05348 | Pamr1 | -2.41162 | 2.67E-07 | Decreased |
| 18251 | Mrc1 | -2.21593 | 2.80E-07 | Decreased |
| 06778 | Mmp19 | -2.0235 | 2.82E-07 | Decreased |
| 17416 | Ppic | -1.52074 | 2.82E-07 | Decreased |
| 20679 | Icam1 | -1.21445 | 2.82E-07 | Decreased |
| 08934 | Tmem65 | -1.73709 | 3.02E-07 | Decreased |
| 39666 | Srpx | -1.92153 | 3.17E-07 | Decreased |
| 08749 | Col5a1 | -1.81272 | 3.18E-07 | Decreased |
| 03622 | Cybb | -1.70504 | 3.19E-07 | Decreased |
| 37765 | Lims1 | -1.27115 | 3.30E-07 | Decreased |
| 16294 | Cd4 | -1.25789 | 3.35E-07 | Decreased |
| 16294 | Cd4 | -1.25789 | 3.35E-07 | Decreased |
| 16119 | Fzd7 | -1.69804 | 3.36E-07 | Decreased |
| 19211 | Olfml3 | -1.5033 | 3.43E-07 | Decreased |
| 18282 | Gda | -2.05309 | 3.46E-07 | Decreased |
| 20482 | Nfatc4 | -1.54205 | 3.78E-07 | Decreased |
| 12840 | Sparc | -1.06723 | 4.66E-07 | Decreased |
| 18659 | Csf1 | -1.43781 | 4.83E-07 | Decreased |
| 20836 | Rorc | -1.38126 | 5.05E-07 | Decreased |
| 03873 | Cpd | -1.5517 | 5.31E-07 | Decreased |
| 26661 | Hcar1 | -2.99429 | 5.38E-07 | Decreased |
| 45992 | Tlr8 | -2.39119 | 5.44E-07 | Decreased |
| 26965 | Tmem140 | -1.57412 | 5.72E-07 | Decreased |
| 16419 | Pdlim5 | -1.18168 | 5.83E-07 | Decreased |
| 39890 | Abcg3l3 | -2.0331 | 5.90E-07 | Decreased |
| 39890 | Abcg3l3 | -2.0331 | 5.90E-07 | Decreased |
| 51235 | C2 | -1.58664 | 6.02E-07 | Decreased |
| 17065 | Arhgap28 | -1.68753 | 6.19E-07 | Decreased |
| 08671 | AABR07019383.1 | -1.17856 | 6.30E-07 | Decreased |
| 21155 | Ctsk | -1.90557 | 6.66E-07 | Decreased |
| 13572 | Lxn | -1.59363 | 6.71E-07 | Decreased |
| 20309 | Gfra3 | -1.81437 | 7.16E-07 | Decreased |
| 33531 | Cacna2d1 | -1.42646 | 7.19E-07 | Decreased |
| 21199 | Fcgr1a | -1.59379 | 7.38E-07 | Decreased |
| 21199 | Fcgr1a | -1.59379 | 7.38E-07 | Decreased |
| 05639 | Ar | -3.09103 | 8.09E-07 | Decreased |
| 10977 | Igfbp6 | -1.977 | 8.36E-07 | Decreased |
| 05478 | Fkbp9 | -1.38513 | 8.65E-07 | Decreased |
| 01807 | Sspn | -2.80434 | 8.78E-07 | Decreased |
| 25120 | Gli1 | -2.16046 | 9.13E-07 | Decreased |
| 15177 | Sun2 | -1.04924 | 9.42E-07 | Decreased |
| 50349 | Mylk4 | -3.58208 | 9.72E-07 | Decreased |
| 08990 | Amotl1 | -1.90672 | 1.05E-06 | Decreased |
| 10997 | Ednrb | -1.63107 | 1.12E-06 | Decreased |
| 45729 | AC117058.1 | 6.616031 | 1.12E-06 | Increased |
| 47046 | Plin4 | -2.76332 | 1.14E-06 | Decreased |
| 10617 | Scube1 | -1.81416 | 1.28E-06 | Decreased |
| 26415 | Col14a1 | -2.38387 | 1.28E-06 | Decreased |
| 46254 | Adgre1 | -1.79564 | 1.31E-06 | Decreased |
| 07202 | Sema3d | -2.17961 | 1.35E-06 | Decreased |
| 05931 | Cpq | -1.50853 | 1.43E-06 | Decreased |
| 46546 | LOC103689954 | -1.86368 | 1.79E-06 | Decreased |
| 57139 | Myoz3 | -3.2467 | 1.82E-06 | Decreased |
| 32788 | Dysf | -1.11525 | 1.83E-06 | Decreased |
| 05413 | Creb3l1 | -1.39917 | 2.02E-06 | Decreased |
| 13917 | Igsf10 | -2.06363 | 2.04E-06 | Decreased |
| 09867 | Tgfb3 | -1.5634 | 2.20E-06 | Decreased |
| 04554 | Dcn | -2.05512 | 2.40E-06 | Decreased |
| 24818 | Eva1b | -1.29564 | 2.48E-06 | Decreased |
| 04645 | Galnt5 | -2.28304 | 2.54E-06 | Decreased |
| 28930 | Dab2 | -1.69632 | 2.76E-06 | Decreased |
| 16917 | Clcn1 | -2.28076 | 2.97E-06 | Decreased |
| 16366 | Colec12 | -1.61735 | 3.01E-06 | Decreased |
| 04610 | Lum | -2.23002 | 3.03E-06 | Decreased |
| 21526 | Slc25a34 | -2.30665 | 4.02E-06 | Decreased |
| 12826 | Creb3l2 | -1.30714 | 4.04E-06 | Decreased |
| 05126 | Slc66a3 | -2.61137 | 4.58E-06 | Decreased |
| 07706 | Prkaa2 | -1.32556 | 4.74E-06 | Decreased |
| 00655 | Ptprc | -1.94532 | 4.86E-06 | Decreased |
| 10800 | Hadhb | -1.53729 | 5.01E-06 | Decreased |
| 15972 | Ano5 | -3.43169 | 5.03E-06 | Decreased |
| 12134 | Scn4a | -2.09459 | 5.04E-06 | Decreased |
| 48222 | Nlrc5 | -1.55418 | 5.07E-06 | Decreased |
| 49829 | AABR07060872.1 | -2.8614 | 5.34E-06 | Decreased |
| 06060 | Matn2 | -1.7214 | 5.57E-06 | Decreased |
| 24089 | Fndc3b | -1.16817 | 5.58E-06 | Decreased |
| 11054 | Laptm5 | -1.43354 | 5.62E-06 | Decreased |
| 52757 | AABR07049695.3 | -4.07158 | 5.88E-06 | Decreased |
| 00569 | Vsir | -1.23766 | 6.64E-06 | Decreased |
| 04328 | Deptor | -1.43895 | 6.93E-06 | Decreased |
| 38784 | Piezo2 | -2.35365 | 6.97E-06 | Decreased |
| 29342 | Scn7a | -1.54264 | 7.33E-06 | Decreased |
| 16164 | Fcrl2 | -2.61452 | 7.57E-06 | Decreased |
| 53145 | Vwa8 | -1.25332 | 7.90E-06 | Decreased |
| 61779 | Man2b2 | -1.16626 | 8.40E-06 | Decreased |
| 04699 | Fibin | -1.65672 | 8.50E-06 | Decreased |
| 02469 | Trim7 | -1.49751 | 8.53E-06 | Decreased |
| 02469 | Trim7 | -1.49751 | 8.53E-06 | Decreased |
| 02746 | Fstl1 | -1.29317 | 8.72E-06 | Decreased |
| 42189 | Rab31 | -1.07821 | 8.82E-06 | Decreased |
| 05650 | Pgf | -1.48443 | 8.87E-06 | Decreased |
| 11134 | Lama2 | -1.75056 | 9.12E-06 | Decreased |
| 11796 | C1r | -1.71115 | 9.43E-06 | Decreased |
| 08904 | Fli1 | -1.39686 | 9.62E-06 | Decreased |
| 05277 | Ptprv | -3.03203 | 1.02E-05 | Decreased |
| 20991 | Ms4a6a | -2.11178 | 1.03E-05 | Decreased |
| 61316 | Ulbp1 | -2.92086 | 1.07E-05 | Decreased |
| 20837 | Cd300lg | -5.19207 | 1.08E-05 | Decreased |
| 48043 | F2r | -1.35911 | 1.18E-05 | Decreased |
| 50869 | Cebpd | -1.28192 | 1.22E-05 | Decreased |
| 50869 | Cebpd | -1.28192 | 1.22E-05 | Decreased |
| 03134 | Slc4a4 | -1.86477 | 1.25E-05 | Decreased |
| 25209 | Plxnd1 | -1.14484 | 1.29E-05 | Decreased |
| 34191 | Fmo1 | -2.13105 | 1.33E-05 | Decreased |
| 09795 | Nfib | -1.28269 | 1.41E-05 | Decreased |
| 01255 | Mlxip | -1.21596 | 1.44E-05 | Decreased |
| 08678 | Antxr1 | -1.72605 | 1.55E-05 | Decreased |
| 00599 | Lama4 | -1.39834 | 1.65E-05 | Decreased |
| 17249 | Zfp366 | -1.78937 | 1.70E-05 | Decreased |
| 27808 | Lilra5 | -2.62283 | 1.70E-05 | Decreased |
| 00187 | Csf2rb | -1.94355 | 1.73E-05 | Decreased |
| 24159 | Fcer1g | -1.13321 | 1.82E-05 | Decreased |
| 26060 | Arsi | -2.28287 | 1.87E-05 | Decreased |
| 26060 | Arsi | -2.28287 | 1.87E-05 | Decreased |
| 59338 | LOC102549714 | -3.22468 | 1.87E-05 | Decreased |
| 59338 | LOC102549714 | -3.22468 | 1.87E-05 | Decreased |
| 16885 | Klf6 | -1.04937 | 1.92E-05 | Decreased |
| 16575 | Tnfrsf1b | -1.45004 | 2.00E-05 | Decreased |
| 06783 | Neb | -2.1894 | 2.03E-05 | Decreased |
| 31167 | AABR07054319.1 | -1.03294 | 2.04E-05 | Decreased |
| 14867 | Synpo2 | -2.65863 | 2.08E-05 | Decreased |
| 16695 | Mmp2 | -1.89832 | 2.08E-05 | Decreased |
| 21100 | Tnfaip8l2 | -1.14318 | 2.08E-05 | Decreased |
| 59579 | Gpt2 | -1.19206 | 2.11E-05 | Decreased |
| 45829 | Thbs1 | -2.11955 | 2.15E-05 | Decreased |
| 20803 | Meox1 | -1.88558 | 2.18E-05 | Decreased |
| 02680 | Lamc1 | -1.41223 | 2.20E-05 | Decreased |
| 00257 | Smpd3 | -1.2659 | 2.24E-05 | Decreased |
| 10986 | Cmtm7 | -1.30152 | 2.28E-05 | Decreased |
| 33110 | Svep1 | -1.38008 | 2.31E-05 | Decreased |
| 19648 | AABR07068316.1 | -1.74002 | 2.32E-05 | Decreased |
| 14021 | Matn4 | -3.42593 | 2.45E-05 | Decreased |
| 10478 | LOC299282 | -3.02795 | 2.53E-05 | Decreased |
| 23683 | Nog | 2.159758 | 2.60E-05 | Increased |
| 04208 | Crim1 | -1.08004 | 2.61E-05 | Decreased |
| 42516 | AC130146.1 | -3.15748 | 2.70E-05 | Decreased |
| 18220 | Pde4dip | -1.4245 | 2.72E-05 | Decreased |
| 17212 | Spsb1 | -1.37819 | 2.75E-05 | Decreased |
| 23334 | Parp14 | -1.39913 | 2.75E-05 | Decreased |
| 04719 | Pp2d1 | 2.034954 | 2.90E-05 | Increased |
| 19542 | MGC108823 | -1.77626 | 2.98E-05 | Decreased |
| 16292 | Pdzd9 | 1.597722 | 3.01E-05 | Increased |
| 23109 | Icoslg | -1.42112 | 3.14E-05 | Decreased |
| 16413 | Pstpip1 | -1.8043 | 3.15E-05 | Decreased |
| 16334 | Ptbp3 | -1.19489 | 3.25E-05 | Decreased |
| 10208 | Timp1 | -1.69044 | 3.29E-05 | Decreased |
| 10253 | Cd163 | -2.05928 | 3.42E-05 | Decreased |
| 61739 | Klrk1 | -3.10036 | 3.43E-05 | Decreased |
| 14350 | Ccn1 | -1.57351 | 3.48E-05 | Decreased |
| 17020 | Inpp5d | -1.30476 | 3.49E-05 | Decreased |
| 53086 | Selenop | -1.59423 | 3.49E-05 | Decreased |
| 42519 | Peak1 | -1.0008 | 3.57E-05 | Decreased |
| 07062 | Rin3 | -1.27958 | 3.62E-05 | Decreased |
| 01389 | Irs3 | -1.33003 | 3.81E-05 | Decreased |
| 32997 | AY172581.20 | 1.562431 | 3.81E-05 | Increased |
| 22610 | Agbl1 | -2.49271 | 3.84E-05 | Decreased |
| 06548 | Mrc2 | -1.77487 | 3.91E-05 | Decreased |
| 07426 | Tmem64 | -1.21908 | 3.96E-05 | Decreased |
| 08949 | Synpo2l | -2.41404 | 4.07E-05 | Decreased |
| 02730 | Rgs5 | -1.41359 | 4.10E-05 | Decreased |
| 08170 | Jph2 | -2.27986 | 4.27E-05 | Decreased |
| 16606 | Snorc | 5.234654 | 4.29E-05 | Increased |
| 02461 | Nid1 | -1.592 | 4.40E-05 | Decreased |
| 06776 | Smyd1 | -2.37754 | 4.46E-05 | Decreased |
| 26679 | Scn4b | -2.1037 | 4.52E-05 | Decreased |
| 57451 | Itga5 | -1.21109 | 4.62E-05 | Decreased |
| 10635 | Igfbp4 | -1.38469 | 4.72E-05 | Decreased |
| 17421 | Alpk2 | -2.41283 | 4.81E-05 | Decreased |
| 03170 | Nlrp3 | -1.83077 | 4.99E-05 | Decreased |
| 03170 | Nlrp3 | -1.83077 | 4.99E-05 | Decreased |
| 13045 | Setd7 | -1.03542 | 4.99E-05 | Decreased |
| 07457 | Serping1 | -1.23461 | 5.13E-05 | Decreased |
| 20457 | Tacc2 | -1.14673 | 5.21E-05 | Decreased |
| 14424 | RGD1563354 | -2.2763 | 5.27E-05 | Decreased |
| 21316 | Tmem98 | 1.230883 | 5.30E-05 | Increased |
| 39560 | LOC103690116 | -1.72123 | 5.43E-05 | Decreased |
| 07250 | Six4 | -1.76733 | 5.61E-05 | Decreased |
| 18346 | Agtr1a | -2.35053 | 5.69E-05 | Decreased |
| 26842 | Nnt | -1.69099 | 5.77E-05 | Decreased |
| 58193 | Slc27a6 | -4.00768 | 5.84E-05 | Decreased |
| 06384 | Ddx58 | -1.39771 | 5.85E-05 | Decreased |
| 16117 | Myof | -1.48715 | 5.89E-05 | Decreased |
| 08736 | Slamf8 | -1.82393 | 5.96E-05 | Decreased |
| 21412 | Slfn13 | -1.18612 | 6.09E-05 | Decreased |
| 15654 | Ghr | -1.17923 | 6.15E-05 | Decreased |
| 51977 | Mmrn2 | -1.14864 | 6.21E-05 | Decreased |
| 45728 | AABR07033887.1 | -1.28059 | 6.24E-05 | Decreased |
| 07345 | Amot | -1.67959 | 6.61E-05 | Decreased |
| 10643 | Kank2 | -1.31866 | 6.65E-05 | Decreased |
| 47186 | Myot | -1.64715 | 6.77E-05 | Decreased |
| 18145 | Crat | -1.74223 | 6.84E-05 | Decreased |
| 00386 | Pbld1 | -3.45587 | 6.96E-05 | Decreased |
| 03587 | Vegfd | -2.59564 | 6.99E-05 | Decreased |
| 03870 | C1qtnf2 | -1.39027 | 7.10E-05 | Decreased |
| 08587 | Tek | -1.1343 | 7.68E-05 | Decreased |
| 32882 | AY172581.19 | 1.100228 | 7.70E-05 | Increased |
| 03088 | Arhgap31 | -1.05375 | 7.79E-05 | Decreased |
| 00195 | LOC108348048 | -1.31197 | 8.08E-05 | Decreased |
| 11425 | Ptpn3 | -1.30813 | 8.12E-05 | Decreased |
| 12428 | Maf | -1.26675 | 8.22E-05 | Decreased |
| 06738 | Fbxo32 | -1.41066 | 8.33E-05 | Decreased |
| 15368 | Pdilt | 1.676579 | 8.63E-05 | Increased |
| 17206 | Igfbp5 | -1.44834 | 8.79E-05 | Decreased |
| 31669 | Lpp | -1.66115 | 9.10E-05 | Decreased |
| 08116 | Plpp3 | -1.38055 | 9.22E-05 | Decreased |
| 48472 | Comp | -2.85585 | 9.23E-05 | Decreased |
| 34139 | Lyc2 | -2.90566 | 9.43E-05 | Decreased |
| 34139 | Lyc2 | -2.90566 | 9.43E-05 | Decreased |
| 33564 | Cfd | -2.63822 | 9.53E-05 | Decreased |
| 33564 | Cfd | -2.63822 | 9.53E-05 | Decreased |
| 09980 | Plpp1 | -1.06869 | 1.02E-04 | Decreased |
| 16071 | P3h3 | -1.15463 | 1.03E-04 | Decreased |
| 15911 | Lrp5 | -1.13334 | 1.04E-04 | Decreased |
| 02244 | Pdgfra | -1.06739 | 1.08E-04 | Decreased |
| 23803 | Cmya5 | -2.04782 | 1.09E-04 | Decreased |
| 43044 | Cnn2 | -1.48598 | 1.09E-04 | Decreased |
| 08409 | Myo1f | -1.65258 | 1.09E-04 | Decreased |
| 15941 | Fkbp10 | -1.16729 | 1.10E-04 | Decreased |
| 01804 | Itpr2 | -1.12214 | 1.16E-04 | Decreased |
| 02832 | Slc16a2 | -1.04681 | 1.19E-04 | Decreased |
| 09826 | Bche | -2.32532 | 1.19E-04 | Decreased |
| 14532 | Lbp | -1.60401 | 1.20E-04 | Decreased |
| 12807 | C1qa | -1.17519 | 1.21E-04 | Decreased |
| 10681 | Pitx2 | -1.78212 | 1.22E-04 | Decreased |
| 12721 | Ednra | -1.46783 | 1.23E-04 | Decreased |
| 31535 | Ptgdrl | -1.96876 | 1.23E-04 | Decreased |
| 11419 | Aldh6a1 | -1.02947 | 1.24E-04 | Decreased |
| 29792 | NEWGENE_1308171 | -1.35951 | 1.29E-04 | Decreased |
| 03172 | Serpinf1 | -1.6446 | 1.30E-04 | Decreased |
| 27259 | AABR07029661.1 | -1.47503 | 1.31E-04 | Decreased |
| 43451 | Spp1 | -1.96368 | 1.31E-04 | Decreased |
| 51298 | AC141377.3 | 1.116948 | 1.34E-04 | Increased |
| 00836 | Ltb | -1.85353 | 1.34E-04 | Decreased |
| 04249 | Tlr7 | -1.53256 | 1.39E-04 | Decreased |
| 07955 | Timp4 | -1.27852 | 1.43E-04 | Decreased |
| 33618 | Col28a1 | -2.10526 | 1.45E-04 | Decreased |
| 06227 | Ifih1 | -1.09433 | 1.46E-04 | Decreased |
| 27540 | Fam102b | -1.17114 | 1.50E-04 | Decreased |
| 14398 | Scara5 | -2.04921 | 1.51E-04 | Decreased |
| 57832 | Rnf125 | -4.43948 | 1.66E-04 | Decreased |
| 52873 | Npnt | -2.02164 | 1.71E-04 | Decreased |
| 13220 | Arhgap45 | -1.15279 | 1.72E-04 | Decreased |
| 33579 | Glt8d2 | -2.15216 | 1.76E-04 | Decreased |
| 14504 | Il1r1 | -2.10913 | 1.77E-04 | Decreased |
| 11250 | Inmt | -1.68516 | 1.79E-04 | Decreased |
| 22910 | Emcn | -1.3807 | 1.82E-04 | Decreased |
| 20353 | Sh3pxd2a | -1.16062 | 1.86E-04 | Decreased |
| 03947 | Ntn1 | -2.16891 | 1.89E-04 | Decreased |
| 09620 | Cybrd1 | -1.68149 | 1.90E-04 | Decreased |
| 01959 | Mx1 | -1.55976 | 1.90E-04 | Decreased |
| 16848 | Fzd4 | -1.26499 | 1.91E-04 | Decreased |
| 24127 | Atp13a5 | -2.48947 | 1.95E-04 | Decreased |
| 00658 | Acacb | -2.28093 | 1.96E-04 | Decreased |
| 12881 | Fgl2 | -1.74497 | 1.97E-04 | Decreased |
| 16244 | Mical2 | -1.17209 | 1.99E-04 | Decreased |
| 62206 | AABR07044460.2 | 1.104572 | 2.02E-04 | Increased |
| 04192 | Arhgap30 | -1.28575 | 2.02E-04 | Decreased |
| 23576 | Ecrg4 | -1.7747 | 2.05E-04 | Decreased |
| 08680 | Loxl1 | -1.2972 | 2.07E-04 | Decreased |
| 42137 | Sting1 | -1.60929 | 2.08E-04 | Decreased |
| 62101 | Ace | -1.21674 | 2.11E-04 | Decreased |
| 06110 | Jph1 | -1.9527 | 2.11E-04 | Decreased |
| 04708 | Aard | 1.459276 | 2.15E-04 | Increased |
| 05352 | Elf4 | -1.84559 | 2.18E-04 | Decreased |
| 59618 | Scarf1 | -1.23025 | 2.18E-04 | Decreased |
| 19278 | Fsd2 | -1.93148 | 2.21E-04 | Decreased |
| 18915 | Tagap | -1.95081 | 2.23E-04 | Decreased |
| 17716 | Ucp3 | -2.11411 | 2.26E-04 | Decreased |
| 30688 | Lrrc2 | -1.81805 | 2.26E-04 | Decreased |
| 04409 | Sash3 | -1.34122 | 2.30E-04 | Decreased |
| 14426 | Lox | -1.47543 | 2.30E-04 | Decreased |
| 01770 | Ehhadh | -2.19812 | 2.34E-04 | Decreased |
| 46414 | Vwa5a | -1.31939 | 2.34E-04 | Decreased |
| 14441 | Krt16 | 5.780189 | 2.35E-04 | Increased |
| 22593 | Pdpr | -1.14383 | 2.39E-04 | Decreased |
| 05166 | Nhlh1 | 1.195122 | 2.40E-04 | Increased |
| 23972 | Col4a2 | -1.17291 | 2.42E-04 | Decreased |
| 10079 | Ca3 | -1.6998 | 2.44E-04 | Decreased |
| 33215 | RT1-Db1 | -1.60009 | 2.46E-04 | Decreased |
| 13426 | Mrgprf | -1.19719 | 2.48E-04 | Decreased |
| 10966 | Itgb1 | -1.04438 | 2.55E-04 | Decreased |
| 21644 | Slc15a3 | -1.20072 | 2.57E-04 | Decreased |
| 51854 | Enpep | -1.8975 | 2.58E-04 | Decreased |
| 29980 | Zbtb16 | -1.35246 | 2.63E-04 | Decreased |
| 07270 | Il12rb2 | -3.38905 | 2.64E-04 | Decreased |
| 02215 | Mylk | -1.54556 | 2.73E-04 | Decreased |
| 08602 | Steap4 | -1.78267 | 0.000278 | Decreased |
| 21203 | Atl3 | -1.72771 | 2.80E-04 | Decreased |
| 03069 | Cd38 | -1.83889 | 2.81E-04 | Decreased |
| 07654 | Lrig3 | -1.23807 | 2.81E-04 | Decreased |
| 13231 | Ptafr | -1.37226 | 2.82E-04 | Decreased |
| 46658 | Omd | -1.64442 | 2.82E-04 | Decreased |
| 00561 | Pald1 | -1.01399 | 2.86E-04 | Decreased |
| 25868 | Klhl33 | -2.46714 | 2.86E-04 | Decreased |
| 14395 | Gli3 | -1.08192 | 3.14E-04 | Decreased |
| 53055 | Otop3 | 1.898511 | 3.21E-04 | Increased |
| 20948 | Pth1r | -1.43937 | 3.23E-04 | Decreased |
| 14574 | Entpd1 | -1.18148 | 3.24E-04 | Decreased |
| 10947 | Mmp14 | -1.16723 | 3.43E-04 | Decreased |
| 49115 | Ccr5 | -1.45826 | 3.47E-04 | Decreased |
| 55293 | Ptprb | -1.41285 | 3.48E-04 | Decreased |
| 18076 | Fmo5 | -2.94618 | 3.52E-04 | Decreased |
| 09074 | AABR07017902.1 | -2.84796 | 3.52E-04 | Decreased |
| 50190 | Eng | -1.00769 | 3.56E-04 | Decreased |
| 16243 | Casq2 | -1.81813 | 3.61E-04 | Decreased |
| 47714 | Tmem37 | -1.28299 | 3.78E-04 | Decreased |
| 17477 | Mmp23 | -1.49026 | 3.81E-04 | Decreased |
| 61868 | Kbtbd12 | -1.98331 | 3.82E-04 | Decreased |
| 08036 | Dennd4c | -1.03199 | 3.86E-04 | Decreased |
| 10107 | AABR07025295.1 | -1.1115 | 3.92E-04 | Decreased |
| 04400 | Avpr1a | -1.87366 | 3.95E-04 | Decreased |
| 53945 | Daam2 | -1.18246 | 4.05E-04 | Decreased |
| 12238 | Clec3a | -1.55667 | 4.13E-04 | Decreased |
| 07350 | Rac2 | -1.02705 | 4.15E-04 | Decreased |
| 22358 | AABR07026012.1 | -2.04944 | 4.19E-04 | Decreased |
| 28581 | Ccdc138 | 1.061371 | 4.20E-04 | Increased |
| 49437 | Gpc1 | -1.47904 | 4.22E-04 | Decreased |
| 28774 | Vgll3 | -1.95428 | 4.25E-04 | Decreased |
| 15519 | Ces1d | -1.98784 | 4.29E-04 | Decreased |
| 53163 | AABR07050084.1 | 2.840103 | 4.31E-04 | Increased |
| 05707 | Bfsp1 | -3.54744 | 4.31E-04 | Decreased |
| 01499 | Mia | 2.774338 | 4.31E-04 | Increased |
| 08445 | Dact1 | -1.402 | 4.35E-04 | Decreased |
| 18715 | Clec10a | -1.58266 | 4.36E-04 | Decreased |
| 29658 | Rnf213 | -1.09646 | 4.41E-04 | Decreased |
| 20078 | Vstm4 | -1.15444 | 4.53E-04 | Decreased |
| 20706 | Kcnn3 | -1.4468 | 4.56E-04 | Decreased |
| 03400 | Fmo4 | -1.86491 | 4.59E-04 | Decreased |
| 03336 | Mybph | -1.77774 | 4.78E-04 | Decreased |
| 15894 | Dock8 | -1.30719 | 4.80E-04 | Decreased |
| 33192 | Osmr | -1.6466 | 4.87E-04 | Decreased |
| 38960 | RGD1309362 | -1.78994 | 4.88E-04 | Decreased |
| 17146 | Nfatc1 | -1.74287 | 5.00E-04 | Decreased |
| 23465 | Depp1 | -1.4032 | 5.01E-04 | Decreased |
| 32307 | Dsel | -1.06342 | 5.18E-04 | Decreased |
| 02161 | Tlr6 | -1.49092 | 5.19E-04 | Decreased |
| 02161 | Tlr6 | -1.49092 | 5.19E-04 | Decreased |
| 16050 | Fgfr1 | -1.24474 | 5.20E-04 | Decreased |
| 27724 | Plekhf1 | -1.29485 | 5.27E-04 | Decreased |
| 14745 | AABR07026654.1 | -1.05681 | 5.33E-04 | Decreased |
| 21161 | Fermt3 | -1.20398 | 5.35E-04 | Decreased |
| 05008 | Angpt4 | -2.27779 | 5.37E-04 | Decreased |
| 49422 | LOC108348047 | -1.71253 | 5.45E-04 | Decreased |
| 49422 | LOC108348047 | -1.71253 | 5.45E-04 | Decreased |
| 49422 | LOC108348047 | -1.71253 | 5.45E-04 | Decreased |
| 49422 | LOC108348047 | -1.71253 | 5.45E-04 | Decreased |
| 50431 | Aspn | -1.68782 | 5.47E-04 | Decreased |
| 05620 | Lcp2 | -1.11275 | 5.56E-04 | Decreased |
| 46663 | Fcgr2a | -1.29856 | 5.65E-04 | Decreased |
| 46663 | Fcgr2a | -1.29856 | 5.65E-04 | Decreased |
| 46663 | Fcgr2a | -1.29856 | 5.65E-04 | Decreased |
| 46663 | Fcgr2a | -1.29856 | 5.65E-04 | Decreased |
| 04706 | Vit | -2.74033 | 5.66E-04 | Decreased |
| 11971 | C1s | -1.62691 | 5.75E-04 | Decreased |
| 08902 | Pon1 | 1.065401 | 5.77E-04 | Increased |
| 19270 | P2ry6 | -1.2541 | 5.82E-04 | Decreased |
| 00459 | Psmb9 | -1.0215 | 5.91E-04 | Decreased |
| 01989 | Alcam | -1.05175 | 5.95E-04 | Decreased |
| 47218 | Clic5 | -1.75171 | 5.97E-04 | Decreased |
| 25881 | Rbms3 | -1.95785 | 6.01E-04 | Decreased |
| 00704 | Cmklr1 | -1.80368 | 6.03E-04 | Decreased |
| 26902 | Lyve1 | -1.64042 | 6.04E-04 | Decreased |
| 53232 | Ror2 | -1.59347 | 6.05E-04 | Decreased |
| 39596 | Prob1 | -1.10126 | 6.07E-04 | Decreased |
| 49811 | LOC684871 | -1.77233 | 6.09E-04 | Decreased |
| 05955 | Naalad2 | -2.41946 | 6.12E-04 | Decreased |
| 37082 | Mybphl | -1.3484 | 6.12E-04 | Decreased |
| 05975 | Rpl30 | 1.704609 | 6.16E-04 | Increased |
| 20956 | Bcat2 | -1.65791 | 6.45E-04 | Decreased |
| 16103 | Nkd2 | -1.3321 | 6.46E-04 | Decreased |
| 23085 | Pmel | -1.58238 | 6.75E-04 | Decreased |
| 45785 | LOC100359687 | 1.000252 | 6.82E-04 | Increased |
| 20300 | Lsp1 | -1.05053 | 6.85E-04 | Decreased |
| 17803 | Apbb1ip | -1.09571 | 6.95E-04 | Decreased |
| 61379 | C7 | -2.21843 | 6.96E-04 | Decreased |
| 11063 | Dennd1b | -1.27809 | 6.98E-04 | Decreased |
| 60614 | Pxdn | -1.47224 | 7.07E-04 | Decreased |
| 31785 | Krt76 | 4.780247 | 7.10E-04 | Increased |
| 50000 | AABR07034739.1 | -3.74021 | 7.18E-04 | Decreased |
| 01296 | P2rx7 | -1.90519 | 7.22E-04 | Decreased |
| 47977 | Tcim | -1.27433 | 7.29E-04 | Decreased |
| 10438 | Cpt1b | -1.66748 | 7.43E-04 | Decreased |
| 10906 | Ccl5 | -2.18863 | 7.49E-04 | Decreased |
| 17767 | Mrvi1 | -1.36798 | 7.68E-04 | Decreased |
| 17767 | Mrvi1 | -1.36798 | 7.68E-04 | Decreased |
| 08941 | Ets1 | -1.16929 | 7.75E-04 | Decreased |
| 45997 | Chst14 | -1.12063 | 7.80E-04 | Decreased |
| 18505 | Cidea | -2.43959 | 7.85E-04 | Decreased |
| 17277 | Igsf6 | -1.73649 | 7.98E-04 | Decreased |
| 36762 | Itpripl2 | -2.03248 | 8.09E-04 | Decreased |
| 10691 | Cmtm3 | -1.20127 | 8.12E-04 | Decreased |
| 30676 | AABR07044373.1 | 7.556767 | 8.14E-04 | Increased |
| 02414 | Tfcp2l1 | -2.04002 | 8.23E-04 | Decreased |
| 09471 | Epsti1 | -1.10676 | 8.29E-04 | Decreased |
| 60179 | Gpc3 | -1.43894 | 8.59E-04 | Decreased |
| 45684 | LOC100910978 | -1.56269 | 8.72E-04 | Decreased |
| 07102 | Acss1 | -1.30519 | 8.85E-04 | Decreased |
| 20865 | Ano1 | -1.90619 | 8.90E-04 | Decreased |
| 14343 | Anln | -1.90207 | 9.06E-04 | Decreased |
| 12216 | Tgfbi | -1.08354 | 9.15E-04 | Decreased |
| 14956 | Slc11a1 | -1.53493 | 9.21E-04 | Decreased |
| 00552 | Col13a1 | -1.02659 | 9.25E-04 | Decreased |
| 49918 | Lrg1 | -1.33013 | 9.28E-04 | Decreased |
| 12068 | LOC685849 | -3.36159 | 9.30E-04 | Decreased |
| 15567 | Slc9a2 | -1.91642 | 9.36E-04 | Decreased |
| 27030 | Adm | -1.0647 | 9.39E-04 | Decreased |
| 21433 | Arhgef39 | 1.488942 | 9.50E-04 | Increased |
| 07839 | Slc16a7 | -1.16635 | 9.71E-04 | Decreased |
| 19365 | Ablim3 | -1.10111 | 9.77E-04 | Decreased |
| 02413 | Gpc4 | -1.38229 | 9.82E-04 | Decreased |
| 03221 | Myoc | -1.62772 | 9.90E-04 | Decreased |
| 29939 | Gypc | -1.42262 | 0.001003 | Decreased |
| 32884 | Scn11a | 2.621616 | 0.001005 | Increased |
| 15505 | Mfap5 | -1.65338 | 0.001006 | Decreased |
| 02372 | Sgcd | -1.86651 | 0.001008 | Decreased |
| 12865 | Parp3 | -1.16979 | 0.001025 | Decreased |
| 40205 | Zcchc24 | -1.1523 | 0.001027 | Decreased |
| 49425 | Fam167b | -1.71477 | 0.001029 | Decreased |
| 15415 | Rhoq | -1.12915 | 0.00103 | Decreased |
| 00383 | Mypn | -1.6464 | 0.001038 | Decreased |
| 18603 | Carns1 | -1.34377 | 0.001042 | Decreased |
| 17912 | Atp2a3 | -1.14733 | 0.001044 | Decreased |
| 08346 | Itgb6 | -1.57574 | 0.001044 | Decreased |
| 14610 | Anpep | -1.57824 | 0.00107 | Decreased |
| 02303 | Kcnj12 | -1.0354 | 0.001073 | Decreased |
| 02303 | Kcnj12 | -1.0354 | 0.001073 | Decreased |
| 55751 | P3h2 | -1.18113 | 0.001099 | Decreased |
| 02014 | Stap1 | -2.73539 | 0.001109 | Decreased |
| 06723 | Itga11 | -1.38706 | 0.001118 | Decreased |
| 56038 | Ehbp1l1 | -1.11468 | 0.00117 | Decreased |
| 03526 | Sytl4 | -2.30314 | 0.001171 | Decreased |
| 11504 | Akap2 | -1.05799 | 0.001178 | Decreased |
| 27115 | Zc2hc1c | -1.49925 | 0.001207 | Decreased |
| 04149 | Mgat4c | 1.034768 | 0.001213 | Increased |
| 09705 | Lck | -1.684 | 0.001254 | Decreased |
| 29191 | LOC685067 | -2.09936 | 0.001256 | Decreased |
| 29191 | LOC685067 | -2.09936 | 0.001256 | Decreased |
| 10240 | Tent5a | -1.11981 | 0.001282 | Decreased |
| 10685 | Tbx18 | -1.73583 | 0.001289 | Decreased |
| 24028 | Sprr1a | 4.866686 | 0.001292 | Increased |
| 21966 | Il17rd | -1.30981 | 0.001293 | Decreased |
| 12779 | Msr1 | -1.73468 | 0.001296 | Decreased |
| 06802 | Lrrn1 | -1.18049 | 0.001313 | Decreased |
| 56457 | Gpd1 | -1.31591 | 0.001362 | Decreased |
| 17403 | Apobr | -1.20257 | 0.001366 | Decreased |
| 29318 | Tyrp1 | -1.07159 | 0.001379 | Decreased |
| 57701 | Myom1 | -1.79824 | 0.00138 | Decreased |
| 08118 | Sync | -1.26274 | 0.001386 | Decreased |
| 00394 | Srgn | -1.03212 | 0.001389 | Decreased |
| 04489 | Adgre5 | -1.4179 | 0.001406 | Decreased |
| 03705 | Bmx | -1.82799 | 0.001409 | Decreased |
| 09197 | Asb4 | -2.50165 | 0.00142 | Decreased |
| 49893 | LOC100910934 | -1.86709 | 0.00143 | Decreased |
| 02408 | Rbm47 | -1.36253 | 0.001442 | Decreased |
| 22999 | Ppp2r3a | -1.12494 | 0.001459 | Decreased |
| 50042 | Myh8 | -3.32277 | 0.001461 | Decreased |
| 22067 | Tlr5 | -2.2684 | 0.00148 | Decreased |
| 23257 | Adamts9 | -1.43774 | 0.001513 | Decreased |
| 02791 | Bcl2 | -1.43902 | 0.001514 | Decreased |
| 58039 | Acta2 | -1.35877 | 0.001521 | Decreased |
| 43350 | Cap2 | -1.0013 | 0.001522 | Decreased |
| 19659 | Aspa | -1.44637 | 0.001535 | Decreased |
| 17105 | Dpyd | -1.64025 | 0.001564 | Decreased |
| 30763 | Dpp4 | -1.99759 | 0.001565 | Decreased |
| 42771 | Apol3 | -1.13518 | 0.001576 | Decreased |
| 09369 | Tor4a | -1.05197 | 0.001581 | Decreased |
| 05906 | LOC103690020 | -3.39013 | 0.001586 | Decreased |
| 60899 | AABR07040840.1 | -1.69602 | 0.001596 | Decreased |
| 07590 | Eya1 | -1.06423 | 0.001628 | Decreased |
| 07159 | Ccl2 | -2.32495 | 0.001634 | Decreased |
| 07159 | Ccl2 | -2.32495 | 0.001634 | Decreased |
| 15310 | Lrrc32 | -1.14505 | 0.001696 | Decreased |
| 23463 | Parp9 | -1.03594 | 0.001764 | Decreased |
| 37134 | Shc4 | -1.62854 | 0.001801 | Decreased |
| 12723 | Trim55 | -1.71836 | 0.001802 | Decreased |
| 15113 | Mocos | -2.21961 | 0.001829 | Decreased |
| 14202 | Snx20 | -1.29117 | 0.001879 | Decreased |
| 09157 | Fut4 | -1.48205 | 0.001879 | Decreased |
| 20583 | Fcgrt | -1.11819 | 0.001893 | Decreased |
| 17773 | Crispld1 | -1.77464 | 0.001909 | Decreased |
| 12966 | Acadl | -1.47902 | 0.001925 | Decreased |
| 27008 | Igtp | -1.00974 | 0.001927 | Decreased |
| 27008 | Igtp | -1.00974 | 0.001927 | Decreased |
| 22704 | Esyt3 | -1.49988 | 0.001931 | Decreased |
| 11758 | Fkbp7 | -1.00633 | 0.001949 | Decreased |
| 02403 | Niban1 | -1.67083 | 0.001962 | Decreased |
| 11478 | Ackr4 | -1.81563 | 0.001988 | Decreased |
| 22800 | Sp140 | -1.13261 | 0.001989 | Decreased |
| 02382 | Mfap4 | -1.28399 | 0.001992 | Decreased |
| 04899 | Kcns3 | -1.10918 | 0.002006 | Decreased |
| 05690 | Lmcd1 | -1.1238 | 0.002024 | Decreased |
| 11044 | Clmn | -1.11595 | 0.002049 | Decreased |
| 24190 | Rasef | -3.17568 | 0.002066 | Decreased |
| 08839 | Pparg | -2.76992 | 0.002072 | Decreased |
| 18906 | Ghdc | -1.00355 | 0.002092 | Decreased |
| 14837 | Emilin2 | -1.79083 | 0.002128 | Decreased |
| 00614 | Bicc1 | -1.66765 | 0.002128 | Decreased |
| 20244 | Perm1 | -1.63796 | 0.00213 | Decreased |
| 09399 | Fads2l1 | -2.25122 | 0.002131 | Decreased |
| 11016 | Slc7a2 | -1.26026 | 0.002148 | Decreased |
| 24433 | Fbxl7 | -1.28952 | 0.002149 | Decreased |
| 18205 | Ttl | -1.17525 | 0.002169 | Decreased |
| 12471 | Thbs4 | -1.60845 | 0.002169 | Decreased |
| 19565 | Tbx15 | -1.71081 | 0.002184 | Decreased |
| 08697 | Ccn3 | -1.26412 | 0.002199 | Decreased |
| 20530 | Cnfn | 8.530734 | 0.002216 | Increased |
| 28996 | Krt1 | 4.228444 | 0.00222 | Increased |
| 06418 | Samhd1 | -1.95622 | 0.002231 | Decreased |
| 06715 | Ccr1 | -1.87308 | 0.002262 | Decreased |
| 01480 | Ncf1 | -1.08178 | 0.002263 | Decreased |
| 17637 | Fbp2 | -1.37431 | 0.002276 | Decreased |
| 37815 | Acad10 | -1.15524 | 0.00228 | Decreased |
| 55934 | Dmkn | 3.312155 | 0.002311 | Increased |
| 51179 | Vps25 | 3.429565 | 0.002317 | Increased |
| 28992 | Acan | 3.168795 | 0.00232 | Increased |
| 28992 | Acan | 3.168795 | 0.00232 | Increased |
| 54957 | Sfrp4 | -2.26292 | 0.002335 | Decreased |
| 04635 | Kera | -1.95298 | 0.002354 | Decreased |
| 01271 | Card6 | -1.00024 | 0.002376 | Decreased |
| 02381 | Bmp3 | -1.21958 | 0.002408 | Decreased |
| 01645 | Filip1l | -1.07769 | 0.002415 | Decreased |
| 05679 | Fap | -1.78447 | 0.002428 | Decreased |
| 30389 | Lrrc30 | -2.42943 | 0.002437 | Decreased |
| 42975 | Tmem45a | -1.74724 | 0.002467 | Decreased |
| 07552 | Arhgap36 | -2.47367 | 0.002478 | Decreased |
| 15406 | Pgm5 | -1.44527 | 0.002502 | Decreased |
| 02881 | Ddr2 | -1.41895 | 0.002511 | Decreased |
| 08880 | Nrk | -3.59379 | 0.002519 | Decreased |
| 03666 | Jchain | -2.70948 | 0.002523 | Decreased |
| 25300 | Fer1l6 | -2.00845 | 0.002524 | Decreased |
| 33883 | Stard8 | -1.32901 | 0.002526 | Decreased |
| 17093 | Pxdc1 | -1.07851 | 0.002532 | Decreased |
| 13781 | Kcnq5 | -1.63235 | 0.002564 | Decreased |
| 02052 | Ccdc80 | -1.46045 | 0.002602 | Decreased |
| 04712 | Angptl1 | -1.4262 | 0.00262 | Decreased |
| 62144 | AABR07035955.1 | -1.66863 | 0.002633 | Decreased |
| 10296 | Slc7a7 | -2.19267 | 0.002648 | Decreased |
| 38916 | Dram1 | -1.33732 | 0.00268 | Decreased |
| 26951 | Susd5 | -1.42035 | 0.00273 | Decreased |
| 60021 | Txlnb | -1.72516 | 0.002779 | Decreased |
| 02862 | Clcn5 | -1.0435 | 0.002781 | Decreased |
| 09448 | Papln | -1.03604 | 0.002817 | Decreased |
| 31716 | LOC100910978 | -1.62057 | 0.002817 | Decreased |
| 48302 | LOC100910979 | -1.67435 | 0.002837 | Decreased |
| 51619 | Asb2 | -1.2652 | 0.002844 | Decreased |
| 03463 | Srebf1 | -1.02826 | 0.002863 | Decreased |
| 28598 | Edar | -1.78277 | 0.002869 | Decreased |
| 24580 | Mamstr | -1.26101 | 0.00291 | Decreased |
| 25895 | Cavin2 | -1.25389 | 0.002955 | Decreased |
| 02459 | Fbxo40 | -2.06543 | 0.002967 | Decreased |
| 04125 | C4bpb | 1.141766 | 0.002981 | Increased |
| 04125 | C4bpb | 1.141766 | 0.002981 | Increased |
| 15029 | Dbt | -1.01595 | 0.002986 | Decreased |
| 20298 | Bag3 | -1.09847 | 0.003025 | Decreased |
| 00443 | LOC103689965 | -1.07902 | 0.003105 | Decreased |
| 18268 | Hhip | -2.40564 | 0.003119 | Decreased |
| 01547 | Agps | -1.72302 | 0.003135 | Decreased |
| 21787 | Zfp217 | -1.23149 | 0.003151 | Decreased |
| 25691 | Pla2g7 | -1.23541 | 0.003159 | Decreased |
| 51307 | Aoc3 | -1.51609 | 0.003189 | Decreased |
| 50485 | Gas1 | -1.0715 | 0.003214 | Decreased |
| 43486 | Tnfrsf26 | -1.21892 | 0.00324 | Decreased |
| 18952 | Sema3g | -1.02963 | 0.003244 | Decreased |
| 45560 | Gvin1 | -2.02998 | 0.003259 | Decreased |
| 01823 | St6gal1 | -1.88938 | 0.003263 | Decreased |
| 11784 | Mocs1 | -1.26497 | 0.003293 | Decreased |
| 49537 | AABR07021544.1 | -1.25666 | 0.0033 | Decreased |
| 03924 | Pi4k2b | -1.02951 | 0.003353 | Decreased |
| 21726 | Tlr3 | -1.57515 | 0.003397 | Decreased |
| 14125 | Evi2b | -1.52685 | 0.003409 | Decreased |
| 01653 | St3gal6 | -1.31933 | 0.003417 | Decreased |
| 50990 | Glyatl3 | -2.28803 | 0.003417 | Decreased |
| 07990 | Adipor2 | -1.09215 | 0.003438 | Decreased |
| 14465 | Oca2 | -1.80545 | 0.003464 | Decreased |
| 00920 | Phkg1 | -1.75033 | 0.003476 | Decreased |
| 12879 | Fabp3 | -1.48979 | 0.003531 | Decreased |
| 20557 | Ryr1 | -1.23452 | 0.003536 | Decreased |
| 60846 | Morc4 | -1.04215 | 0.003556 | Decreased |
| 26371 | Krt17 | 3.83803 | 0.003585 | Increased |
| 10763 | Fam181b | -1.43653 | 0.003601 | Decreased |
| 20308 | Ech1 | -1.13036 | 0.003675 | Decreased |
| 17528 | Gpr157 | -1.02862 | 0.003686 | Decreased |
| 51570 | AABR07001910.1 | -1.00424 | 0.003695 | Decreased |
| 02093 | Tgfbr3 | -1.10256 | 0.003724 | Decreased |
| 49942 | RGD1564899 | -1.47089 | 0.003768 | Decreased |
| 22711 | Slco4c1 | -1.90415 | 0.003823 | Decreased |
| 04687 | Thbd | -1.08637 | 0.00383 | Decreased |
| 58340 | Krt79 | -1.74552 | 0.003838 | Decreased |
| 06946 | Arhgap9 | -1.71516 | 0.003864 | Decreased |
| 55049 | Aldh1a2 | -1.11631 | 0.003878 | Decreased |
| 18991 | Gsn | -1.49139 | 0.003924 | Decreased |
| 12660 | Postn | -1.12333 | 0.00396 | Decreased |
| 22353 | Klk13 | 11.22905 | 0.004052 | Increased |
| 08182 | Htra3 | -1.21371 | 0.004071 | Decreased |
| 31058 | Was | -1.24093 | 0.004101 | Decreased |
| 04720 | Kcnj2 | -1.49358 | 0.004122 | Decreased |
| 16281 | Col4a1 | -1.19052 | 0.004127 | Decreased |
| 06543 | Bsnd | -1.54982 | 0.004198 | Decreased |
| 03956 | Arhgap6 | -1.65371 | 0.004236 | Decreased |
| 21294 | Mstn | -1.05088 | 0.004266 | Decreased |
| 07545 | Angptl4 | -1.22838 | 0.004269 | Decreased |
| 01571 | Cldn8 | -2.43156 | 0.004295 | Decreased |

# Supplementary Fingure


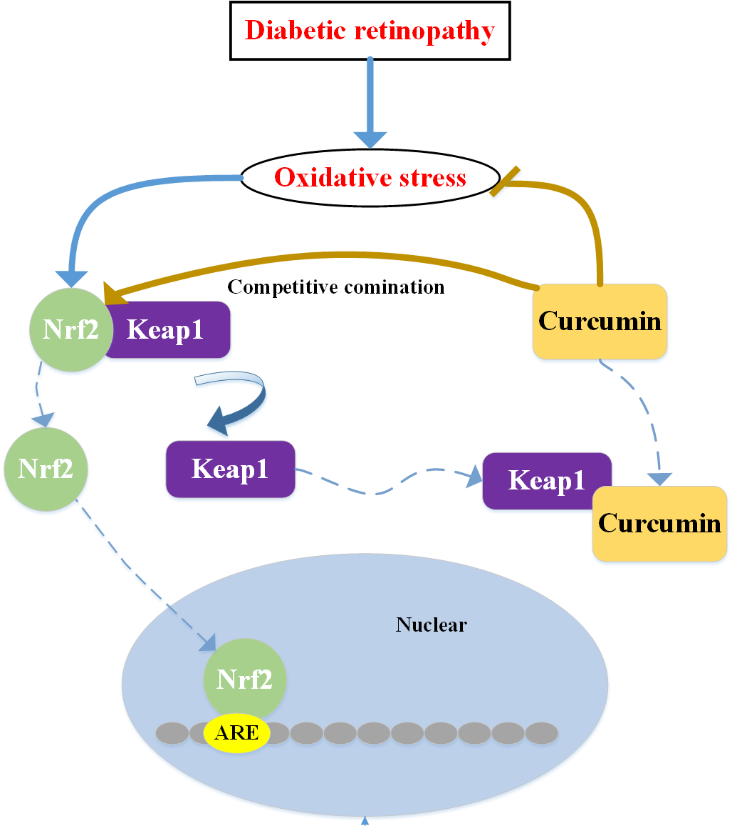


**Figure S1**. Proposed mechanism in the introduction
